# Identification of a VPS29 isoform with restricted association to Retriever and Retromer accessory proteins through autoinhibition

**DOI:** 10.1073/pnas.2501111122

**Published:** 2025-06-30

**Authors:** James L. Daly, Kai-en Chen, Rebeka Butkovič, Qian Guo, Michael D. Healy, Eva Pennink, Georgia Gamble-Strutt, Zara Higham, Edmund R. R. Moody, Philip A. Lewis, Kate J. Heesom, Tom A. Williams, Kirsty J. McMillan, Brett M. Collins, Peter J. Cullen

**Affiliations:** ^a^School of Biochemistry, Faculty of Health and Life Sciences, University of Bristol, Bristol BS8 1TD, United Kingdom; ^b^School of Immunology and Microbial Sciences, Faculty of Life Sciences and Medicine, Guy’s Hospital, King’s College London, London SE1 9RT, United Kingdom; ^c^Institute for Molecular Bioscience, The University of Queensland, St. Lucia, QLD 4072, Australia; ^d^School of Biological Sciences, University of Bristol, Bristol BS8 1TD, United Kingdom; ^e^Bristol Proteomics Facility, School of Biochemistry, Faculty of Life Sciences, University of Bristol, Bristol BS8 1TD, United Kingdom; ^f^Department of Biochemistry, Cell and Systems Biology, Institute of Systems, Molecular and Integrative Biology, University of Liverpool, Liverpool L69 3BX, United Kingdom

**Keywords:** Retromer, Retriever, Commander, endosomes, lysosomes

## Abstract

The endosomal–lysosomal network is a hub of organelles that orchestrate the dynamic sorting of hundreds of integral membrane proteins to maintain cellular homeostasis. VPS29 is a central conductor of this network through its assembly into Retromer, Retriever, and Commander endosomal sorting complexes, and its role in regulating RAB GTPase activity. Two VPS29 isoforms have been described, VPS29A and VPS29B, that differ solely in their amino-terminal sequences. Here, we identify a third VPS29 isoform, which we term VPS29C, that harbors an extended amino-terminal sequence compared to VPS29A and VPS29B. Through a combination of AlphaFold predictive modeling, in vitro complex reconstitution, mass spectrometry, and molecular cell biology, we find that the amino-terminal VPS29C extension constitutes an autoinhibitory sequence that limits access to a hydrophobic groove necessary for effector protein recruitment to Retromer, and association with Retriever and Commander. VPS29C is therefore unique in its ability to uncouple Retromer-dependent cargo sorting from the broader roles of VPS29A and VPS29B in regulating the endosomal–lysosomal network through accessory protein recruitment. Our identification and characterization of VPS29C points to additional complexity in the differential subunit assembly of Retromer, an important consideration given the increasing interest in Retromer as a potential therapeutic target in neurodegenerative diseases.

## Significance

The endosomal–lysosomal network is essential for normal cellular function with network defects being associated with numerous neurodegenerative diseases. Two heterotrimeric complexes, Retromer and Retriever, control transmembrane protein recycling through the network. Of these, reduced Retromer expression is observed in Alzheimer's disease and Retromer mutations lead to familial Parkinson's disease. Here, we identify and characterize an isoform of VPS29, a subunit shared between Retromer and Retriever. We reveal how this isoform, VPS29C, adopts an auto-inhibitory conformation to limit association into Retriever and restrict binding of VPS29C-containing Retromer to accessory proteins vital for regulating network function. By revealing added complexity in Retromer assembly, we provide insights into how Retromer function influences endosomal sorting and maturation that underpin neurodegenerative disease.

Across eukaryotes the establishment, maintenance, and adaptation of the functional cell surface proteome requires the selective sorting of internalized integral proteins through the intracellular endosomal network (1–3). The efficient sorting of channels, enzymes, transporters, receptors, adhesion molecules, and polarity cues is essential for an array of cellular, tissue, and organism-level processes and physiology, with sorting defects being linked to developmental and age-related human diseases including cancer, metabolic syndromes, and neurodegenerative diseases (4–7).

Upon entering the endosomal network integral proteins and associated lipids and proteins (termed “cargo”) are sorted between two fates; either degradation within the lysosome or retrieval from this fate for recycling and reuse at the cell surface and other intracellular organelles (3). While Endosomal Sorting Complex Required for Transport (ESCRT) proteins regulate the degradative fate (8) a series of multiprotein assemblies orchestrate the retrieval and recycling pathways (9, 10). These include the Retromer, Endosomal SNX-BAR Sorting Complex Promoting Exit-1 (ESCPE-1), and Wiskott Aldrich Syndrome Protein and Scar Homologue (WASH) complexes (11–16), the Retriever, COMMD–CCDC22–CCDC93 (CCC) and Commander assemblies (17–21), and additional sorting nexins (SNXs), SNX-Bin-Amphiphysin-Rvs (BAR)s, and other proteins including ACAP1 (22–33).

Retromer and Retriever are stable heterotrimers assembled with a similar blueprint. For Retromer the amino-terminal end of the core VPS35 α-solenoid associates with VPS26A or VPS26B while its carboxy-terminal end binds to VPS29 (34). Similarly, the amino-terminal end of the Retriever VPS35L α-solenoid binds to VPS26C, and the carboxy-terminal region binds to VPS29 (19–21). Retromer and Retriever, therefore, share VPS29 as a common subunit.

VPS29 adopts a phosphoesterase-fold and contains two conserved hydrophobic patches (35, 36). One associates with the carboxy-terminal regions of the VPS35 and VPS35L α-solenoids (19–21, 37), the other is solvent exposed and contains a surface groove. In Retromer, this groove is the primary site for binding to a variety of accessory proteins including TBC1D5, ANKRD27, and the FAM21 subunit of the WASH complex, that each present Pro-Leu motifs to occupy the groove (14, 38–48). In the case of Retriever, the VPS29 hydrophobic groove is occupied by an intramolecular Pro-Leu motif from the extended unstructured amino-terminal region of VPS35L (19–21). This intramolecular interaction stabilizes the Retriever heterotrimer and its association with the CCC complex within the Commander supercomplex and acts to prevent Retriever from binding to Retromer accessory proteins (19, 49). The function of the shared VPS29 subunit between Retromer and Retriever is therefore context dependent (34).

Two human VPS29 isoforms have been identified that differ solely in their amino terminal sequences. The shorter form comprises ^1^MLVLVL^6^ (here termed VPS29A), while the longer form arises through inclusion of an additional short exon by alternative splicing and encodes four additional amino acids to give the sequence ^1^MAGHRLVLVL^10^ (here termed VPS29B). Both isoforms can assemble into Retromer but it has been suggested that the ^2^AGHR^5^ insertion modulates the affinity of VPS29 binding to the effector proteins TBC1D5 and AKNRD27 (42, 45, 46). Here, we identify a third human VPS29 isoform, VPS29C, with a significantly extended amino-terminal sequence of 28 amino acids. Using a combination of AlphaFold modeling, in vitro biochemical reconstitutions, and molecular cell biology approaches, we reveal that VPS29C preferentially assembles into the Retromer over the Retriever heterotrimer. We show that this stems from the 28-amino acid amino-terminal extension in VPS29C forming a transient intramolecular association that inhibits the hydrophobic groove necessary for VPS29 assembly into Retriever. In addition, this intramolecular association results in a VPS29C–Retromer complex that displays a limited ability to associate with Retromer accessory proteins that also depend on this VPS29 surface. Our identification and characterization of VPS29C points to additional complexity in the differential subunit assembly of Retromer, an important consideration given the increasing interest in Retromer as a potential therapeutic target in neurodegenerative diseases (50).

## Results

### Identification of a Third VPS29 Splice Isoform.

**Fig. 1.**
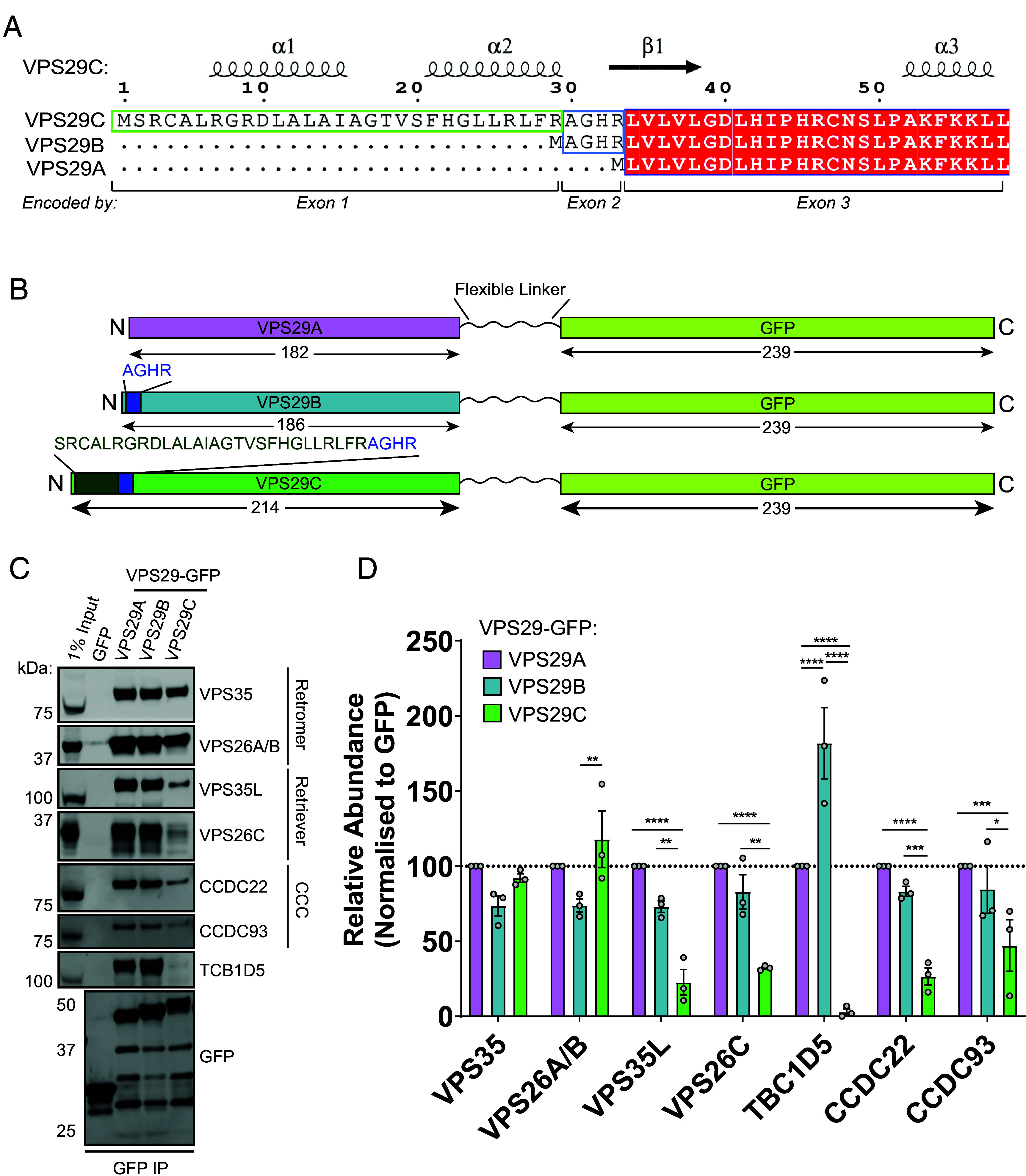
Identification of a VPS29 isoform that assembles into a functional Retromer complex. (*A*) Protein sequence alignment and corresponding exon junctions of human VPS29 splice isoforms reveal a unique amino-terminal extension loop in VPS29C. (*B*) Construct map describing the design of VPS29A/B/C-GFP constructs, with amino terminal extensions of VPS29B and VPS29C highlighted, and a flexible linker separating the carboxy-terminus of VPS29 from the amino-terminus of GFP. (*C*) GFP-nanotrap immunoprecipitation of VPS29A/B/C-GFP isoforms interaction with Retromer, Commander, and TBC1D5. (*D*) Quantification of relative protein abundances in (*C*). Means ± SEM, n = 3 independent experiments two-way ANOVA with Tukey’s multiple comparisons tests. *P < 0.05, **P < 0.01, ***P < 0.001, ****P < 0.0001.

Two VPS29 isoforms, which we here term VPS29A and VPS29B, are ubiquitously expressed in human tissues and have been characterized at the molecular level (12, 35, 45, 46, 51). These isoforms are generated by alternative splicing of the *VPS29* gene. We further noted a third computationally mapped alternative splicing variant of human *VPS29* (herein referred to as VPS29C, Gencode ID: ENST00000546588.1, UniProt ID: F8VXU5), which encodes an amino-terminal extension of 28 amino acids immediately prior to the ^2^AGHR^5^ insertion of VPS29B (Fig. 1*A*).

To confirm the endogenous expression of VPS29C in human cells, we prepared complementary DNA (cDNA) from HeLa and HEK293T cells, along with Caco-2 (colon adenocarcinoma), Calu-3 (lung adenocarcinoma), and induced pluripotent human stem cells (iPSCs) differentiated into cortical neurons to assess tissue distribution. By performing PCRs using 5′ end primers recognizing either *VPS29* exon 3 (shared by all VPS29 isoforms), exon 2 (shared by VPS29B and VPS29C), or exon 1 (unique to VPS29C) and a 3′ end primer shared by all isoforms, we could identify transcripts corresponding to the predicted sizes of *VPS29A* (546 bp), *VPS29B* (558 bp), and *VPS29C* (645 bp) in all cell lines (*SI Appendix*, Fig. S1*A*). *VPS29C* levels were considerably lower than *VPS29A/B* levels in all cells tested. To unambiguously confirm the identity of the VPS29C band, we extracted and sequenced the PCR product from HeLa and HEK293T cells, which exactly matched the consensus sequence for *VPS29C* (*SI Appendix*, Fig. S1 *B* and *C* and *Supporting Information Text*).

An additional bioinformatic search revealed that highly similar extensions are found in a selection of primates, and more distantly related sequences are also present in several other mammalian lineages. This suggests that the VPS29C isoform likely evolved in the common ancestor of placental mammals after the divergence of placental mammals from marsupials (*SI Appendix*, Fig. S2 *A* and *B* and Dataset S1).

### VPS29C Exhibits Altered Binding Properties to VPS29A/B.

To compare the biological functions of these VPS29 isoforms, VPS29A, VPS29B, and VPS29C were carboxy-terminally tagged with green fluorescent protein (GFP) to leave their respective amino-terminal sequences unperturbed (Fig. 1*B*). Expression of VPS29A/B/C-GFP constructs in HEK293T cells followed by GFP-trap immunoprecipitation to isolate interacting proteins revealed a conserved ability of all three isoforms to associate with components of Retromer (VPS26A/B and VPS35) (Fig. 1 *C* and *D*). VPS29A and VPS29B both robustly immunoprecipitated the well-documented VPS29 effector protein TCB1D5 with VPS29B showing a stronger association in agreement with in vitro biochemical data (38, 42, 44–46). Moreover, VPS29A and VPS29B are also associated with components of the Retriever complex and Commander superassembly with which VPS29 is known to interact (18–21). In comparison, VPS29C demonstrated significantly perturbed binding to the Retriever subunits VPS26C and VPS35L as well as components of the Commander complex CCDC22 and CCDC93. Moreover, VPS29C binding to TBC1D5 was also severely impaired (Fig. 1 *C* and *D*).

### Interactome Analysis Is Consistent with VPS29C Displaying Reduced Association to Accessory Proteins and Incorporation into Retriever.

**Fig. 2.**
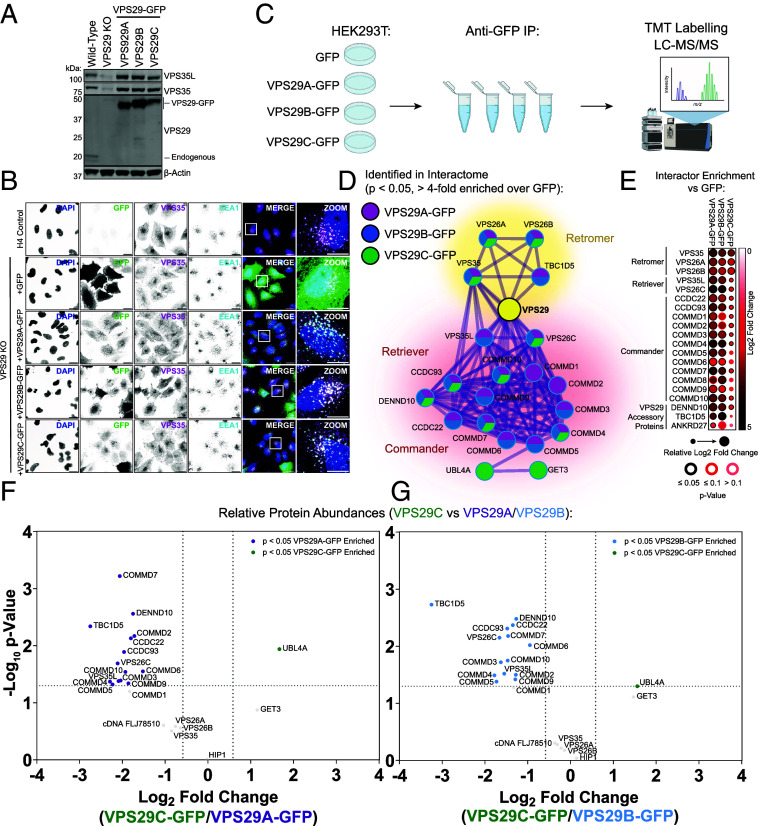
VPS29 isoforms exhibit different profiles of protein–protein interactions. (*A*) Stable H4 neuroglioma cell lines were made by rescuing VPS29 KO clonal cells with VPS29A/B/C-GFP. (*B*) Immunofluorescence microscopy of wild-type H4 cells and VPS29 KO H4 cells transfected with GFP, VPS29A-GFP, VPS29B-GFP, or VPS29C-GFP. Cells were costained with anti-VPS35 and anti-EEA1 antibodies. (Scale bar, 20 µm, *Inset* scale bar, 10 µm.) (*C*) Schematic of mass spectrometry experimental design. Figure created with Biorender.com (*D*) Protein–protein interaction network of the isolated interactomes of VPS29A/B/C-GFP. Proteins are colored by which VPS29-GFP isoform they were significantly identified by (Log_2_ fold change over GFP > 2, *P* < 0.05) as indicated in the legend. (*E*) Dotplots displaying relative enrichment of VPS29A/B/C-GFP interactors over GFP. Dot color indicates Log_2_ fold change relative to GFP, dot size indicates relative fold change between VPS29 isoforms, and border colors indicate *P* value. (*F* and *G*) Volcano plots displaying the relative abundances of significant VPS29 interactors between isoforms following normalization to GFP expression levels. Comparisons are made between VPS29C vs. VPS29A (*F*) and VPS29C vs. VPS29B (*G*) Significantly enriched or depleted proteins (Log_2_ fold change > 0.5, *P* < 0.05) are colored.

To expand our analysis, we performed an unbiased quantitative identification of the comparative VPS29A, VPS29B, and VPS29C interactomes. To establish this procedure, we first validated the expression and localization of VPS29A/B/C constructs, each tagged with GFP at their carboxy termini, by rescuing a previously validated VPS29 knockout (KO) H4 neuroglioma cell line using CRISPR-Cas9 guide RNAs that target all three VPS29 isoforms (52) (Fig. 2*A*). Immunofluorescence analysis revealed that all three isoforms retained association with endosomes where they colocalized with endogenous VPS35 and the early endosomal marker EEA1 (Fig. 2*B*).

We next expressed these constructs in HEK293T cells and performed GFP-trap immunoprecipitation followed by tandem mass spectrometry (TMT)-based proteomics to identify interactors of the three VPS29 isoforms (Fig. 2*C*). 19, 18, and 13 proteins were identified as significantly enriched by VPS29A-GFP, VPS29B-GFP, and VPS29C-GFP, respectively (defined as proteins with >Log_2_ twofold enrichment over GFP-only condition, *P* < 0.05) (Fig. 2*D*, *SI Appendix*, Fig. S3 *A*–*C* and Table S1, and Dataset S2). These interactors included almost all previously validated interactors of VPS29, including components of the Retromer, Retriever, and Commander complexes, and TBC1D5 (Fig. 2 *D* and *E*). In agreement with our biochemical data, VPS29C robustly interacted with the Retromer components VPS35, VPS26A, and VPS26B, but demonstrated weaker associations with all other interactors including Retriever, the Commander complex, TBC1D5 (Fig. 2 *D* and *E*). The association between VPS26C and ANKRD27 was also reduced, although ANRKD27 was only quantified in two experimental replicates (Fig. 2*E*). In addition, UBL4A and GET3 were identified as uniquely enriched by VPS29C compared to VPS29A/B (Fig. 2*D* and *SI Appendix*, Fig. S3*C*). Notably, transmembrane Retromer and Retriever cargo proteins were not identified by any of the VPS29 isoforms, in line with previous findings of Retromer subunit immunoprecipitation combined with mass spectrometry (53). This is likely due to the requirement for the coincidence detection of cargo, sequence-specific adaptors such as sorting nexin (SNX) proteins, and membrane lipid identity for Retromer and Retriever to robustly engage their transmembrane cargo.

To compare between VPS29 isoforms, the proteomics dataset was filtered for the proteins significantly interacting with at least one VPS29 isoform as defined in Fig. 2*D*, then the abundances of interacting proteins were normalized to GFP, to control for differences in VPS29-GFP expression levels between conditions (*SI Appendix*, Table S2 and Dataset S3). The relative enrichments of proteins between VPS29 isoforms were then assessed, revealing that VPS29C-GFP displayed a significantly reduced association with almost all components of the Retriever and Commander complexes (VPS35L, VPS26C, CCDC22, CCDC93, COMMD1-10, and DENND10), and TBC1D5 when compared to VPS29A/B-GFP (<Log_2_ 0.5-fold change, *P* < 0.05) (Fig. 2 *F* and *G*) (19–21, 38, 42, 44–46). Direct comparisons of significant interactors revealed minor differences between the interactomes of VPS29A-GFP and VPS29B-GFP, with VPS29B-GFP demonstrating a stronger enrichment of TBC1D5 in line with previous data and our quantitative western blotting (Fig. 1 *C* and *D*), although this did not reach statistical significance here (*SI Appendix*, Fig. S3*D*). Taken together, these data define an interaction network for all three VPS29 isoforms and confirm that VPS29C demonstrates a reduced capacity to engage Retriever and associated Commander subunits, and most known Retromer interactors besides the core Retromer complex.

### Structural Modeling of VPS29C Reveals an Extended Helical Amino Terminus.

**Fig. 3.**
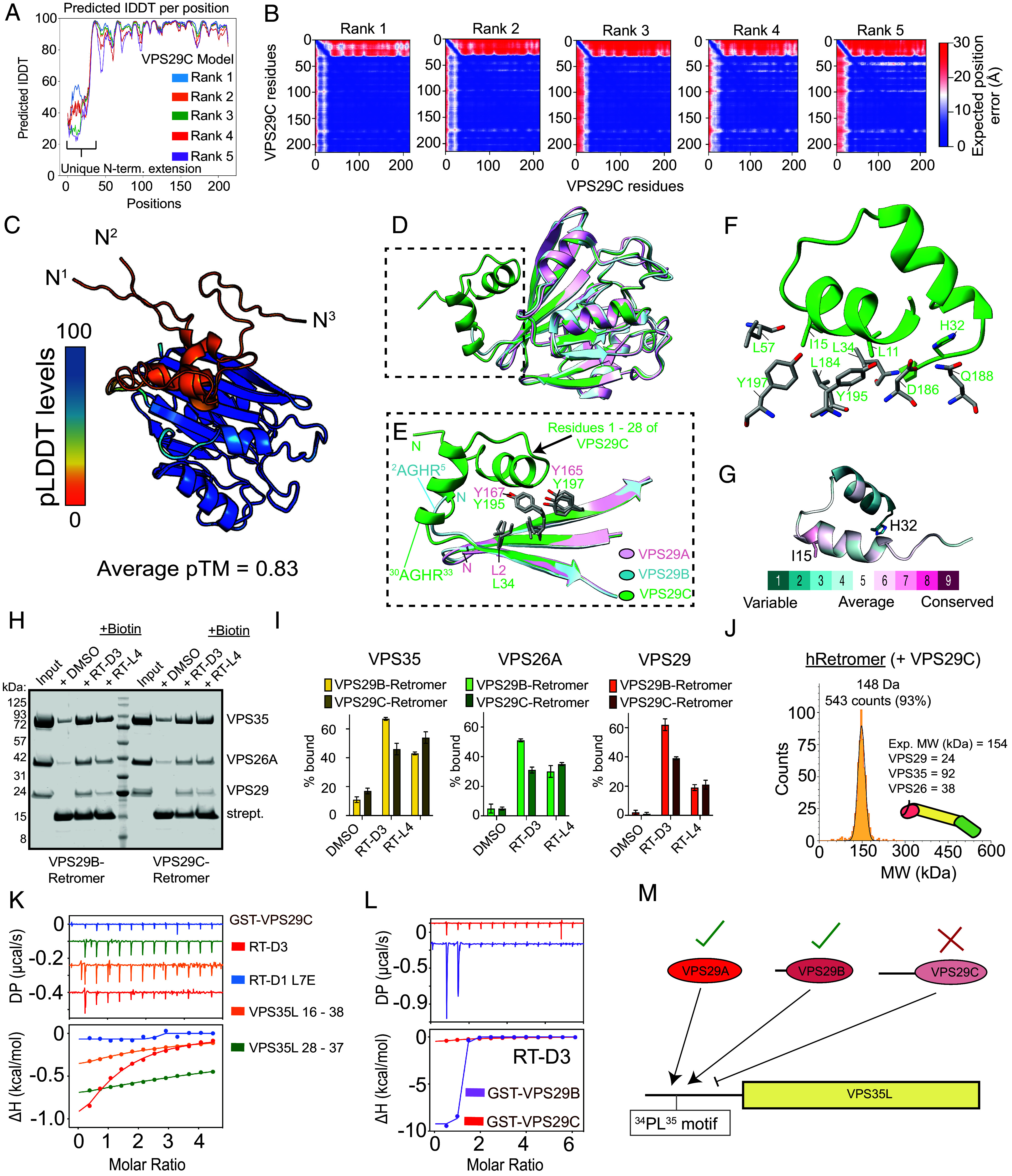
VPS29C displays an altered binding profile to other VPS29 isoforms. (*A*) AlphaFold2 pLDDT plot and (*B*) PAE plot of human VPS29C highlighting the flexibility of the extension loop. (*C*) Cartoon representation of AlphaFold2 predicted human VPS29C model. Three top ranked models are overlaid and colored according to the pLDDT score. (*D*) Superposition of top ranked VPS29C model with the crystal structures of VPS29A (PDB ID: 5OSI) and VPS29B (PDB ID: 5GTU) to highlight the difference of the amino-terminal extension. (*E*) Highlight of the key binding region between the conserved hydrophobic pocket and the extension loop of VPS29C. (*F*) Highlight of the key interacting residues (in stick) of VPS29 shown in (*E*). (*G*) Sequence conservation map showing the conserved residues within the extension loop. (*H*) Pull-down assay of human Retromer containing VPS29B or VPS29C with streptavidin agarose coated with biotinylated RT-D3 or RT-L4. (*I*) Quantitation of the streptavidin-based pull-down assay shown in (*H*). Light yellow, green, and red indicates the samples from VPS29B-containing Retromer. The heavy/dark yellow, green, and red indicates the samples from VPS29C-containing Retromer, n = 2. (*J*) Mass photometry showing VPS29C-containing Retromer forming a 1:1:1 ratio complex. (*K*) ITC measurements of GST-VPS29C with RT-D3 (red), RT-D1 L7E (blue), and two different VPS35L Pro-Leu motif containing peptides (orange and green respectively). (*L*) ITC measurements of RT-D3 with either GST-VPS29B or GST-VPS29C to highlight the impact of the extension loop on binding to the Pro-Leu motif containing macrocyclic peptide known to show strong affinity to human VPS29A/B. All ITC graphs show the integrated and normalized data fit with a 1:1 ratio. (*M*) Schematic diagram summarizing the binding capacity of each VPS29 isoform for VPS35L.

To investigate the cause of the diminished VPS29C interaction network, we modeled the structure of VPS29C using AlphaFold2 implemented in ColabFold. The predicted local distance difference test (pLDDT) scores of 30 to 40 for the unique amino-terminal extension suggest that the region is predominantly unstructured (Fig. 3*A*). A similar prediction was also observed in models generated by AlphaFold3 and RoseTTAFold2 (*SI Appendix*, Fig. S4 *A* and *B*). Further analysis of the predicted aligned error (PAE) plot and multiple models generated from different seeds revealed that the unique amino-terminal extension is commonly predicted to form a short helical stretch positioned in proximity to the flat β-sheet surface of the VPS29 core (Fig. 3 *B* and *C* and *SI Appendix*, Fig. S4 *A* and *B*). Alignment of this model with previously solved X-ray crystallographic structures of VPS29A and VPS29B revealed minimal deviation of the VPS29C model besides this amino-terminal extension (Fig. 3 *D* and *E*). The hydrophobic groove bound by the VPS29C amino-terminus is known to mediate binding to the Retromer accessory proteins TBC1D5, ANKRD27, and FAM21 (42, 45–48), as well as the bacterial protein RidL (54–56). This same pocket is also essential for VPS29 incorporation into the Retriever complex, through binding to amino-terminal sequences in the VPS35L subunit (19–21).

Looking more closely at the VPS29C model, we found the amino-terminal extension was typically oriented toward the VPS29 core due to the ^2^AGHR^5^ sequence forming a U-turn conformation as observed in the VPS29B structure (Fig. 3*E*) (45). In three out of five models predicted by AlphaFold2, the hydrophobic residues within the amino-terminal extension including Leu11 and the conserved Ile15 reinforce this conformation through contact with Leu184 in the hydrophobic groove, which is equivalent to the Leu152/156 residue in VPS29A/B respectively that mediates accessory protein binding (Fig. 3 *F* and *G*). We next compared the predicted structures of extended VPS29 isoforms from other mammalian species. While primate models appeared almost identical to the human VPS29C prediction in length and conformation (*SI Appendix*, Fig. S5*A*), models from more distantly related mammals displayed variation in the length and structure of the amino-terminal extension yet were still regularly predicted to fold back over the hydrophobic groove in a similar way (*SI Appendix*, Fig. S5*B*). These predictive models therefore suggest that the amino-terminal extension in VPS29C may form an intramolecular association with the potential to occlude access to the hydrophobic groove of VPS29 that is essential for binding to Retromer accessory proteins and in stabilizing the Retriever heterotrimer.

### Recombinant VPS29C Displays Perturbed Ligand Binding.

To validate that VPS29C could assemble into a Retromer heterotrimer, we first expressed and purified recombinant VPS35, VPS26A and VPS29B or VPS29C. Based on our previously published methods for purifying VPS29A- and VPS29B-containing Retromer complexes, we could similarly isolate the VPS29C-containing Retromer assembly (57). We next used a streptavidin-based pull-down using two biotin-tagged cyclic peptides known to associate with Retromer as the probe (57). For each cyclic peptide their incubation with the purified fractions revealed successful binding of all three proteins consistent with the formation of a stable VPS29C-containing Retromer heterotrimer complex (Fig. 3 *H* and *I*). The nature of the VPS29C–Retromer complex was further examined by mass photometry which revealed a single peak with a molecular weight of 148 kDa, close to the expected molecular weight of Retromer (153.8 kDa) forming a 1:1:1 stoichiometric ratio complex (Fig. 3*J*). Together, these independent biochemical approaches establish that VPS29C can efficiently assemble into a heterotrimeric Retromer complex like the VPS29A- and VPS29B-containing Retromer, supporting our immunoprecipitation data.

As discussed previously, VPS29A and VPS29B play crucial roles in the Retromer complex by dynamically recruiting effector proteins that modulate the dynamics of endosomal maturation such as TBC1D5, ANKRD27, and the WASH complex through FAM21 (38, 44–48), and in the Retriever complex by engaging the amino-terminus of the core VPS35L subunit (19–21). In these examples, binding occurs at a hydrophobic interface comprising a leucine residue (Leu-152 in VPS29A, Leu-156 in VPS29B), associated with a Pro-Leu motif in the interacting protein. In this mechanism, the ligand proline residue kinks the amino acid chain for optimal leucine engagement with the VPS29A/B binding pocket (*SI Appendix*, Fig. S6). The Vps5 subunit of the *Saccharomyces cerevisiae* pentameric Retromer complex also engages Vps29 through this binding mechanism, indicating that this hydrophobic interface is evolutionarily conserved (*SI Appendix*, Fig. S6) (58, 59). Given that the corresponding hydrophobic interface in VPS29C appears to be at least partially occupied by the extended amino-terminus, we next investigated whether this may constitute an autoinhibitory mechanism that precludes ligand binding.

While we successfully isolated the VPS29C–Retromer using the streptavidin-based pull-down with high affinity cyclic peptides, we observed a subtle difference compared to the VPS29B–Retromer. Of the two cyclic peptides we used, RT-D3 presents a PL motif that specifically associates with high affinity to the Leu-containing hydrophobic groove in VPS29, such that it can outcompete binding to Retromer accessory proteins requiring this site, while the cyclic peptide RT-L4 binds to a region spanning the VPS26 interface with VPS35, without affecting the PL motif containing proteins from binding to VPS29 (57). RT-L4 was able to isolate both VPS29B–Retromer and VPS29C–Retromer equally, whereas RT-D3 was less efficient at isolating VPS29C–Retromer (Fig. 3 *H* and *I*). The reduced ability of RT-D3 to bind VPS29C may therefore be a consequence of the extended amino-terminus precluding access to Leu-184, while binding to other proteins such as VPS35 at distal interfaces is unaffected.

We further validated the ability of VPS29C to engage ligands by utilizing isothermal titration calorimetry (ITC) to directly explore the binding affinity of cyclic peptides and natural peptides corresponding to the amino-terminal region of VPS35L, which contains a Pro-Leu motif that engages the VPS29 leucine pocket in the Retriever assembly (19–21). We observed binding of VPS29C to RT-D3 with a Kd of 13.4 μM while the RT-D1 L7E mutant control peptide, which we previously demonstrated has dramatically reduced affinity for VPS29, showed no binding as expected (Fig. 3*K*). Although the PL motif-containing RT-D3 cyclic peptide can interact with VPS29C it has a significantly lower affinity compared to VPS29B (13.4 μM Kd for VPS29C vs. 0.014 µM Kd for VPS29B) (Fig. 3 *K* and *L* and *SI Appendix*, Table S3). Consistent with these results and coimmunoprecipitation experiments we also found that VPS35L peptides harboring a Pro-Leu motif showed only a weak binding affinity for VPS29C with a Kd > 100 µM compared to the VPS29A data published previously (Fig. 3 *K* and *L* and *SI Appendix*, Table S3) (19). Overall, these experiments demonstrate the unique amino-terminal extension of VPS29C diminishes its ability to engage VPS35L when compared to VPS29A/B (Fig. 3*M*).

### Mechanistic Basis of VPS29C Autoinhibition through an Intramolecular Interaction.

**Fig. 4.**
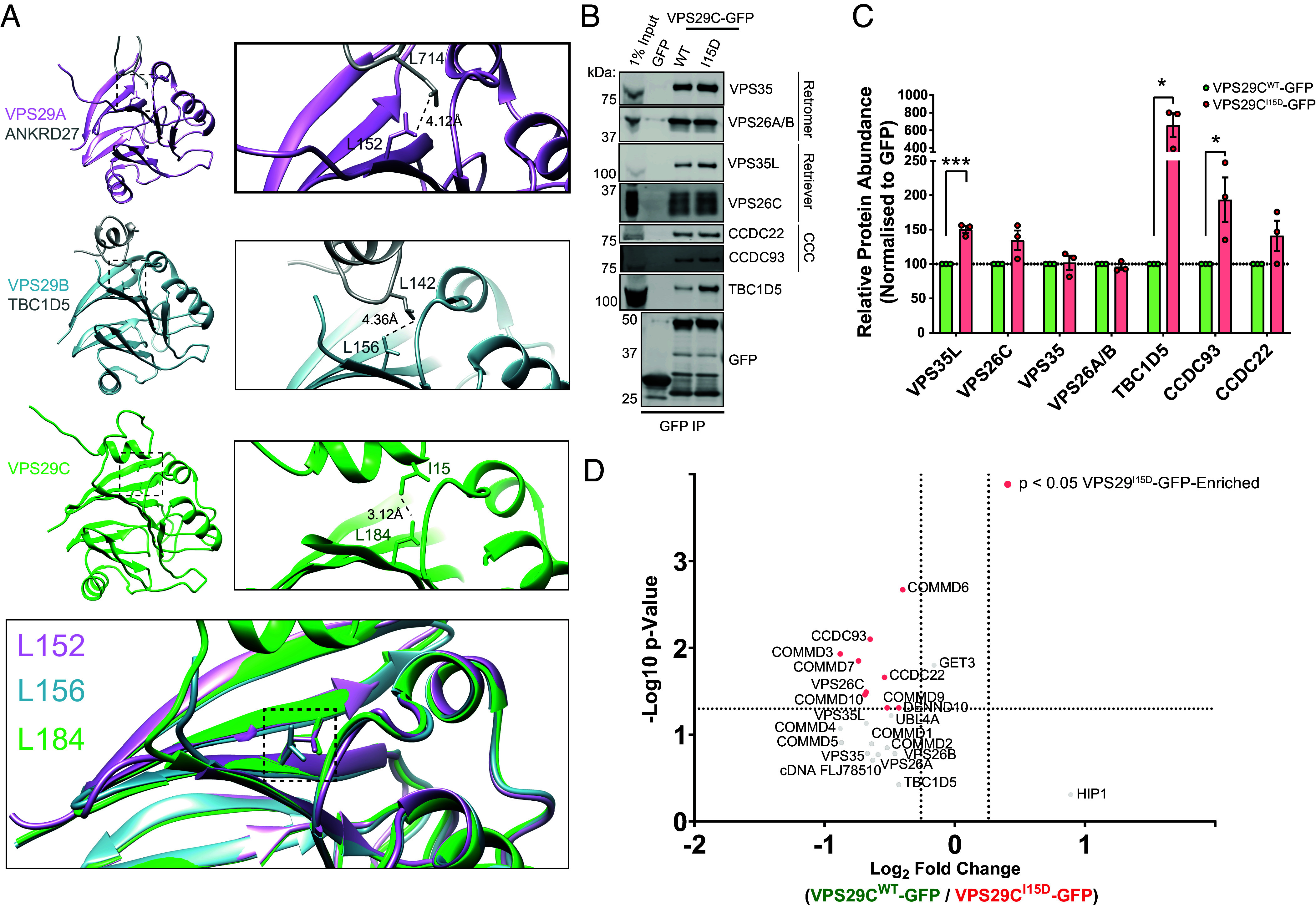
VPS29C is autoinhibited by an intramolecular interaction. (*A*) Comparison of VPS29A and VPS29B structures in complex with interacting regions of ANKRD27 (PDB: 6TL0) and TBC1D5 (PDB: 5GTU) respectively, with the VPS29C intramolecular interaction (AlphaFold model), and an overlay. The enlarged panels demonstrate the Leu152 (VPS29A) Leu156 (VPS29B) and Leu184 (VPS29C) residues involved in the binding interfaces. (*B* and *C*) GFP-nanotrap VPS29C^WT^-GFP and VPS29C^I15D^-GFP constructs expressed in HEK293T cells, followed by quantitative immunoblotting for VPS29 interactors. Means ± SEM, n = 3 independent experiments, unpaired *t* test. *P < 0.05, **P < 0.01, ***P < 0.001, ****P < 0.0001. (*D*) Volcano plots displaying the relative abundances of significant VPS29 interactors between VPS29C^WT^-GFP and VPS29C^I15D^-GFP following normalization to GFP expression levels, n = 3 independent experiments.

Our examinations of VPS29C structural models identified hydrophobic residues, such as Leu-11 and Ile-15, that fold back toward Leu-184 in a conformation that resembles VPS29 ligand binding (Figs. 3 *D*–*G* and 4*A*). The proximity of these hydrophobic residues may constitute an autoinhibitory mechanism that prevents the binding partners of VPS29 from accessing Leu-184, hence explaining the diminished binding of VPS29C to TBC1D5 and ANKRD27, and Retriever and Commander subunits (Fig. 2*E*).

To test this hypothesis, we generated a VPS29C-GFP mutant with Ile-15 mutated to Asp (VPS29^I15D^) to introduce electrostatic charge into this hydrophobic sequence. Comparison of VPS29^WT^-GFP and VPS29^I15D^-GFP immunoprecipitations in HEK293T cells revealed an enhanced capacity for the mutant form to engage a selection of proteins, including significant enrichment of VPS35L, CCDC93, and TBC1D5, while binding to the core Retromer subunits VPS26A and VPS35 was unaffected (Fig. 4 *B* and *C*). Furthermore, quantitative proteomic comparisons of VPS29^WT^-GFP and VPS29^I15D^-GFP interactors confirmed that this restoration of binding extends more widely to components of the Commander complex indicating a broader restoration of binding capacity, though TBC1D5 enrichment did not reach statistical significance through this approach (Fig. 4*D*). Together these data are consistent with the amino-terminal extension of VPS29C serving to block access to the hydrophobic Leu-184-containing interaction interface.

### VPS29C Fails to Regulate RAB7 Dynamics and Endosomal–Lysosomal Morphology.

**Fig. 5.**
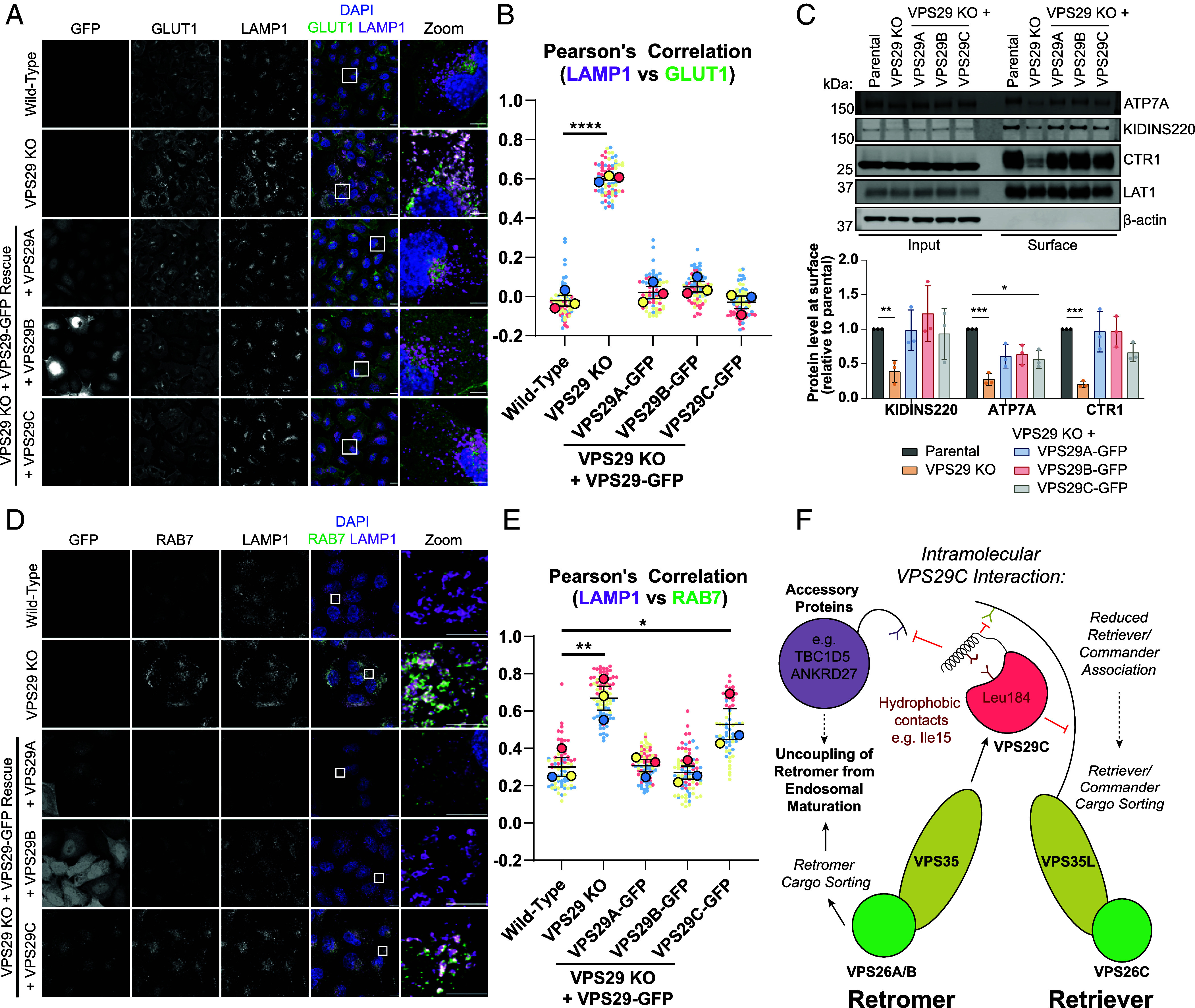
VPS29C-GFP can facilitate Retromer cargo sorting but is unable to regulate RAB7 Activity. (*A*) Immunofluorescence labeling of GLUT1 and LAMP1 in WT, VPS29 KO, and VPS29A/B/C-GFP-expressing H4 cells. (Scale bar, 20 µm.) (*B*) Quantification of Pearson’s correlation between GLUT1 and LAMP1. One-way ANOVA with Dunnett’s multiple comparisons tests, n = 3 independent experiments, *P < 0.05, **P < 0.01, ***P < 0.001, ****P < 0.0001. (*C*) Surface biotinylation and streptavidin affinity isolation of endogenous transmembrane surface cargoes in VPS29 KO and rescue cells, n = 3 independent experiments. Two-way ANOVA with Dunnett’s multiple comparisons test. (*D*) Immunofluorescence labeling of RAB7 and LAMP1 in WT, VPS29 KO, and VPS29A/B/C-GFP-expressing H4 cells. (Scale bar, 20 µm.) (*E*) Quantification of Pearson’s correlation between RAB7 and LAMP1. One-way ANOVA with Dunnett’s multiple comparisons tests, n = 3 independent experiments, *P < 0.05, **P < 0.01, ***P < 0.001, ****P < 0.0001. (*F*) Model for the autoinhibitory function of the VPS29C amino-terminus in dictating Retromer and Retriever function.

To explore the functional consequences of the altered associations of VPS29C, we utilized a VPS29 KO H4 neuroglioma clone stably rescued with VPS29A/B/C-GFP constructs (Fig. 2*A*). We recently characterized the widespread changes to endosomal–lysosomal morphology, identity, and dynamics that occur because of deletion of the Retromer subunits VPS35 or VPS29 in H4 cells (52). We therefore investigated the ability of the VPS29-GFP constructs to reverse these phenotypes, beginning with cargo sorting. The glucose transporter GLUT1 is an established SNX27-Retromer cargo that undergoes endosome-to-plasma membrane trafficking through tubulovesicular carriers generated by the endosomal sorting complex for promoting exit-1 (ESCPE-1) (15, 16, 32, 60, 61). In VPS29 KO H4 cells, GLUT1 is strongly rerouted from the cell surface into LAMP1-positive compartments in the absence of sequence-dependent cargo sorting (Fig. 5 *A* and *B*). Strikingly, this phenotype is rescued by reexpression of all three VPS29 isoforms, indicating that VPS29C assembles into a functional Retromer complex that can engage the SNX27 cargo adaptor and the ESCPE-1 complex to sort cargo away from a degradative fate. Furthermore, surface biotinylation confirmed the ability of VPS29C to regulate the sorting of a selection of transmembrane cargo to the cell surface (Fig. 5*C*). Of these, KIDINS220 is a SNX27-Retromer cargo (32); ATP7A trafficking is regulated by SNX27-Retromer and components of the Commander complex (17, 32), and was partially rescued by VPS29C-GFP, though to significantly lower levels than wild-type cells; and CTR1 is a SNX27-independent Retromer cargo (62). ATP7A and CTR1 are trafficked by Retromer through distinct pathways to regulate copper homeostasis. These data therefore establish that VPS29C can sort Retromer cargo through distinct trafficking pathways involving different cargo adaptors.

It has previously been demonstrated that depletion of Retromer leads to RAB7 hyperactivation due to the lack of recruitment of the RAB7-guanosine triphosphatase (GTPase)-activating protein (GAP) and VPS29 binding partner TBC1D5 (63, 64). This is evidenced by the extensive recruitment of RAB7 to swollen LAMP1-positive compartments in VPS29 KO cells (Fig. 5*D*). As expected, reexpression of VPS29A/B-GFP restored this phenotype by limiting RAB7/LAMP1 colocalization and resolving lysosomal morphology to a similar level to wild-type cells (Fig. 5 *D* and *E*) (52). In contrast, VPS29C-GFP, which binds TBC1D5 less efficiently, failed to rescue this phenotype despite its localization on endosomal membranes (Fig. 5 *D* and *E*). VPS29C is therefore able to uncouple the key functions of the Retromer complex as a master regulator of endosomal biology, by mediating cargo sorting but failing to control RAB7 GTP hydrolysis and effective endosomal–lysosomal maturation and resolution (Fig. 5*F*).

## Discussion

Recent studies have illuminated the central role of VPS29 in orchestrating endosomal cargo sorting and dynamics through coordinating the Retromer, Retriever, and Commander complexes (18–21, 45, 63). Here, we identify and characterize the biochemical properties of an additional isoform of VPS29, which we term VPS29C. This isoform contains an extended amino-terminal helical sequence that autoinhibits the hydrophobic groove on the VPS29 surface required for effector protein recruitment and assimilation into the Retriever complex. VPS29C therefore assembles preferentially into the Retromer complex to facilitate sequence-dependent cargo sorting but uncouples this activity from Retromer’s broader role in regulating RAB7 nucleotide cycling by its RAB7 GAP effector TBC1D5. For example, given the established role of the VPS29–TBC1D5–RAB7 interaction in controlling productive autophagy, it is therefore plausible that VPS29C is unable to regulate autophagy compared to VPS29A/B, future work will be required to confirm this hypothesis (63, 65).

Our recent work suggests that this subset of Retromer would therefore be less capable of regulating endosomal maturation, endosomal–lysosomal fusion and lysosomal reformation (52). Moreover, our proteomics data suggest that the extended amino-terminus of VPS29 does not appreciably expand its interaction network through gaining new interaction partners, but rather appears more likely to be autoinhibitory. Our biochemical data supporting this mechanism of autoinhibition, and its reversal through targeted site-directed mutagenesis, serve as an important functional validation of VPS29 activity and how its conducting role within the endosomal network is driven by accessibility of its interaction partners (Fig. 5*F*).

Subunits of the Retromer and Retriever complexes are evolutionarily conserved among all eukaryotes. Notably, other ancient components of the endosomal sorting machinery have diversified into paralogues or isoforms that appear to play species- and tissue-specific roles. The modulation of endosomal sorting complexes through the selective incorporation of subunit isoforms or paralogues may provide context-dependent fine-tuning of endosomal–lysosomal dynamics. For example, alternative splicing of the *CLTC* gene to include an extra exon can modulate a switch between the formation of clathrin-coated pits to plaques in a tissue-specific context (66, 67). Additionally, the ESCPE-1 subunit SNX32 is a paralogue of SNX6 that is predominantly expressed in the brain where it may engage neuronal cargoes (68). Three paralogues of VPS26 are expressed in humans, termed VPS26A, VPS26B, and VPS26C, of which the former two assimilate into Retromer and the latter into Retriever (18, 69, 70). The VPS26B gene emerged in chordates through duplication of VPS26A, whereas VPS26A and VPS26C are conserved among eukaryotes (71). VPS26A and VPS26B predominantly differ in their carboxy-terminal sequences, and while they exhibit almost identical interaction networks in human retinal pigment epithelial cells (53), differential cargo engagement by these proteins has been suggested (71–73). Mutations associated with atypical parkinsonism are found in VPS26A specifically (53, 74) and conversely VPS26B-Retromer is specifically enriched in the *trans*-entorhinal cortex region of the brain that is particularly vulnerable to Alzheimer’s disease-related neurodegeneration (75), emphasizing that spatially coordinated expression of subunit-specific endosomal sorting complexes and engagement of tissue-specific cargoes may be crucial to neuroprotection.

In contrast to the widespread conservation of the Retromer and Retriever complexes, the extended VPS29C amino-terminal sequences appear to have arisen relatively recently in evolutionary history and have apparently been independently lost on a number of occasions. AlphaFold modeling suggests that these mammalian amino-terminal extensions are, like the human sequence, also predicted to fold in over the conserved VPS29 hydrophobic groove in a conformation that resembles the autoinhibited state of VPS29C. These models raise the interesting possibility that the modulation of VPS29 activity through alternative splicing arose following the divergence of placental mammals from marsupials, though substantiating this hypothesis requires future structural and biochemical validation. The tissue expression profiles of VPS29A, VPS29B, and VPS29C remain to be clarified, but it is conceivable that similar subtle differences of VPS29 isoform localization and cargo engagement may occur across tissues. Spatially mapping VPS29C expression therefore represents an avenue of future work that may illuminate its functional context.

In conclusion, this study characterizes an additional VPS29 isoform that regulates effector binding through autoinhibition. The biochemical inhibitory mechanism we elucidate serves to validate the central orchestrating role of VPS29 in the endosomal network. Given that the concerted activity of VPS29-associated endosomal sorting complexes are broadly considered neuroprotective, approaches to modulate their activity represent promising future therapeutic avenues. The autoinhibitory VPS29C isoform we describe here therefore adds further evidence to the notion that activity of these crucial proteins can be fine-tuned and may inform the future development of therapeutic strategies to rebalance Retromer activity.

## Methods

### Materials and Methods.

#### Antibodies.

Primary antibodies include: β-Actin [Sigma-Aldrich; A1978; clone AC-15; 1:2,000 Western blot (WB)], ATP7A (Santa Cruz Biotechnology; sc-376467; WB 1:1000), CCDC22 (Proteintech, 16636-1-AP, 1:1,000 WB), CCDC93 (LS Bio, C336997, 1:1,000 WB), CTR1 (Abcam; ab129067; WB 1:1000), EEA1 [Cell Signalling, 3288S, 1:200 immunofluorescence (IF)], GFP (Roche; 11814460001; clones 7.1/13.1; 1:1,000 WB; 1:400 IF), GLUT1 (Abcam; EPR3915; ab115730; 1:200 IF), KIDINS-220 (Proteintech; 21856-1-AP; 1:1000 WB), LAMP1 (Developmental Studies Hybridoma Bank; AB_2296838; clone H4A3; 1:400 IF), LAMP1 (Abcam; ab21470; 1:200 IF), RAB7 (Abcam; EPR7589; ab137029; 1:200 IF), LAT1 (Cell Signalling Technologies; 5347; 1:1000 WB), TBC1D5 (Abcam, 204896, 1:1,000 WB), VPS26A (Abcam, ab137447, 1:1,000 WB), VPS26C (Merck, ABN87, 1:1,000 WB), VPS29 (Santa Cruz; D-1; sc-398874; 1:500 WB), VPS35 [Abcam; ab157220; clone EPR11501(B); 1:1,000 WB], VPS35 (Abcam, ab10099, 1:200 IF), VPS35L (Abcam, ab97889, 1:1,000 WB)

Secondary antibodies: For western blotting, 680 nm and 800 nm donkey anti-mouse and anti-rabbit fluorescent secondary antibodies (Invitrogen, A-21057, A3275—1:20,000). For immunofluorescence, 488 nm, 568 nm, and 647 nm AlexaFluor-labeled anti-mouse, anti-rabbit and anti-goat secondary antibodies (Invitrogen, A32753, A32731, A10037, A10042, A32787, A-11057- 1:400). 0.5 µg/mL DAPI dihydrochloride (Sigma-Aldrich, D8417) was added to secondary antibody mixtures to label DNA.

#### Cell culture.

HEK293T cells were sourced from the American Type Culture Collection (ATCC). H4 neuroglioma cells were a gift from Helen Scott (University of Bristol, United Kingdom) and James Uney (University of Bristol, United Kingdom). Clonal VPS29 KO cells were generated by transfecting cells with Cas9 and guide RNA sequences targeting the sequences 5′-GGACATCAAGTTATTCCAT-3′ and 5′-GGCAAACTGTTGCACCGGTG-3′ within *VPS29* and validated previously (52).

Cells were grown in Dulbecco’s Modified Eagle Medium (DMEM; Sigma-Aldrich), supplemented with 10% (vol/vol) fetal bovine serum (FBS) (Sigma-Aldrich) and penicillin/streptomycin (Gibco). H4 cells were transduced with HIV-1-based lentiviruses for stable expression (construct of interest in pLVX-GFP-IRES-Puro plasmid backbone, and pCMV-dR8.91 packing plasmid) pseudotyped with vesicular stomatitis virus (VSV)-G envelope plasmid (pMDG2). HEK293T cells were transfected with the constituent plasmids using polyethylenimine (PEI) transfection, then lentiviruses were harvested after 48 h. H4 cells were seeded into a plate, then transduced with lentivirus following adherence. 3 μg/mL puromycin dihydrochloride was used for selection of VPS29-GFP-expressing cells.

#### Synthesis and cloning of VPS29 isoforms.

The protein-coding sequences of VPS29A, VPS29B, and VPS29C followed by a flexible linker encoding the sequence GGGGSGGGGS were synthesized as dsDNA fragments (Eurofins Genomics) with flanking restriction sites to produce the cassette (*EcoRI*)-VPS29-(*KpnI*)-Linker-(*BamHI*), then digested with EcoRI and BamHI and ligated into the GFP-containing backbones pEGFP-N1 for transient transfection, or pLVX-GFP-IRES-Puro for stable lentiviral transduction.

#### RNA extraction, cDNA synthesis, and PCR analysis.

RNA was extracted from HeLa, HEK239T, Caco-2, and Calu-3 cells using the Monarch Total RNA Miniprep Kit with a DNAse I removal step (New England Biolabs). RNA was extracted from neuronal iPSC cultures using phenol chloroform extraction. cDNA was prepared from total RNA extracts using a High-Capacity cDNA Reverse Transcription Kit (Applied Biosystems). To amplify *VPS29* isoforms from cDNA, the following primers were used: an exon 5 reverse primer common to all isoforms (5′-AGGTTTTTTGTATTCGATTCGTTCTACTTTC-3′), an exon 3 forward primer common to all isoforms (5′- TTGGTGTTGGTATTAGGAGATCTGCAC-3′), an exon 2 forward primer shared by *VPS29B* and *VPS29C* (5′-GCTGGGCACAGATTGGTGTTG-3′) and an exon 1 forward primer specific to *VPS29C* (5′-AGCAGGTGTGCTCTCAGAGG-3′). The band corresponding *to VPS29C* was extracted and purified using a QIAquick Gel Extraction Kit (Qiagen). PCR sequencing was performed by Plasmidsaurus using Oxford Nanopore Technology with custom analysis and annotation.

#### Surface biotinylation.

All buffers were prechilled to 4 °C. Cells were washed twice in ice-cold PBS prior to immersion in ice-cold PBS (pH7.7) containing 200 µg/mL EZ-link sulfo-NHS-SS-biotin (Thermo Scientific #A39258) for 30 min with gentle agitation at 4 °C. To remove excess biotin, cells were washed in 1× PBS followed by 1× in Quench buffer (50 mM Tris, 100 mM NaCl, final pH7.5) prior to a 10 min incubation in quench buffer with gentle agitation. Cells were lysed by scraping in PBS (2% TX100 and protease inhibitor tablets) prior to pelleting of insoluble debris by centrifugation (14 k for 10 min). An aliquot of the subsequent cleared lysate was retained to represent the whole cell fraction and the remainder added to prewashed (in lysis buffer) streptavidin beads (Streptavidin sepharose—Cytiva #17511301). Precipitation of biotinylated cell surface proteins proceeded for 30 min at 4 °C, prior to 1× wash in PBS + 1% TX100, 1× wash in PBS + 1% TX100 and 1 M NaCl and a final wash in PBS. Biotin precipitated beads were pelleted by centrifugation and all traces of wash buffer removed prior to subsequent analyses.

#### Quantitative western blotting.

NuPAGE 4 to 12% gradient Bis-Tris precast gels (Life Technologies, NPO336) were used for SDS-PAGE, followed by transfer onto methanol-activated polyvinylidene fluoride (PVDF) membrane (Immobilon-FL membrane, pore size 0.45 μm; Millipore, IPFL00010). Membrane was blocked, then sequentially labeled with primary and secondary antibodies. Fluorescence detected by scanning with a LI-COR CLS Odyssey scanner and Image Studio analysis software (LI-COR Biosciences) was used to quantify band intensities.

#### Immunofluorescence microscopy and analysis.

H4 cells were transfected with GFP or VPS29-GFP constructs using Fugene 6 (Promega) or stably expressed using lentiviral vectors. H4 cells were seeded onto 13 mm coverslips the day before fixation. DMEM was removed, followed by two washes with PBS, then cells were fixed in 4% paraformaldehyde (PFA) (Pierce, 28906) for 20 min at room temperature. Cells were permeabilized in either 0.1% Triton X-100 or 0.1% (w/v) saponin (Sigma-Aldrich, 47036) for 5 min followed by blocking with 1% (w/v) BSA, (plus 0.01% saponin for those permeabilized by saponin) in PBS for 15 min. Coverslips were stained with primary antibodies for 1 h, followed by secondary antibodies for 30 to 60 min, then mounted onto glass microscope slides with Fluoromount-G (Invitrogen, 00-4958-02).

Confocal microscope images were taken on either a Leica SP8 confocal laser scanning microscope attached to a Leica DM l8 inverted epifluorescence microscope or a Leica SP5-II Biosystems confocal laser-scanning microscope attached to a Leica DMI6000 inverted epifluorescence microscope (Leica Microsystems), with a 63× UV oil immersion lens, numerical aperture 1.4 (Leica Microsystems, 506192), and acquired using LAS AF software (Leica Microsystems). Colocalization and fluorescence intensity analysis was performed using Volocity 6.3 software (PerkinElmer) with automatic Costes background thresholding. Immunofluorescence images were prepared in either Image J (Fiji) or Volocity 6.3.

#### GFP immunoprecipitation.

HEK293T cells were transfected with GFP or VPS29-GFP constructs using polyethylenimine (PEI), then lysed 48 h later with lysis buffer [50 mM Tris-HCl, 0.5% NP-40 PBS with 1 × protease inhibitor cocktail (Roche)]. Lysates were spun at 18,000 × g for 10 min at 4 °C, then the supernatants were transferred onto GFP-trap beads (Proteintech, GTA20) and incubated for 1 h at 4 °C. Beads were then washed twice with 50 mM Tris-HCl, 0.25% NP-40 PBS wash buffer, then once with wash buffer without NP-40 prior to elution in 2× LDS sample buffer (Invitrogen), 1.5% β-mercaptoethanol.

#### Recombinant protein expression and purification in bacteria.

For the expression of human Retromer in bacteria, full-length cDNA encoding human VPS35, human VPS26A, human VPS29C, and mouse VPS29B were cloned into either N-terminal His-tagged pET28a or GST-tagged pGEX6P1 vectors.

All the bacterial constructs were expressed in BL21 (DE3) competent cells using the standard autoinduction method as described previously. Cell cultures were harvested by centrifugation at 6,000 × g for 5 min at 4 °C and the cell pellets were then stored at −80 °C until cell lysis. To obtain individual Retromer subunit including VPS26A, GST-VPS29B, and GST-VPS29C, the frozen cell pellet was resuspended in protein buffer (50 mM Tris-HCl pH 7.5, 200 mM NaCl, 2 mM β-mercaptoethanol) supplemented with 50 μg/mL benzamidine, 100 units of DNase I. In all cases, the resuspended cells were lysed through a Constant System TS-series high pressure cell disruptor. The soluble homogenate cleared by centrifugation was loaded onto either Talon resin (Clontech) for His-tagged VPS26A or Glutathione Sepharose (GE Healthcare) for GST-tagged VPS29B/VPS29C. The fusion-tagged containing proteins eluted from the resin were then further purified using size exclusion chromatography (SEC) on a HiLoad® 16/600 Superdex 200 or Superdex 75 column equilibrated with protein buffer. For Retromer, the soluble homogenate was first loaded onto Talon resin (Clontech) followed by glutathione Sepharose (GE healthcare) to obtain the correct stoichiometry ratio of the Retromer complex. Removal of the GST tag from the constructs was performed using an on-column cleavage approach with PreScission protease. The fusion tag free protein was further purified using SEC on a HiLoad® 16/600 Superdex 200 column as described above. Protein integrity was validated using SDS-PAGE gel and concentration was measured using Nanodrop at OD280.

#### Isothermal titration calorimetry.

All microcalorimetry experiments were carried out at 25 °C using a PEAQ ITC (Malvern, UK) in protein buffer. The interaction of VPS29 and peptides were carried out by titrating 300 μM of RT-D3, RT-D1 L7E, VPS35L 28 to 37 or VPS35L 16 to 38 into 10 μM of GST-tagged VPS29C or GST-tagged VPS29B. In the macrocyclic peptide experiment, the equivalent percentage (v/v) of DMSO was added into the target protein to avoid buffer mismatch. All ITC experiments were performed with a single injection of 0.4 μL followed by a series of 12 injections of 3.2 μL each, spaced 180 s apart, and stirred at 750 rpm. To calculate the heat exchange of interactions, the observed peaks were integrated with the subtraction of heat of dilution from the background. The thermodynamic parameters including Kd, ΔH, ΔG, and −TΔS were obtained by fitting and normalizing the data to a single-site binding model using Malvern software package. The stoichiometry was adjusted initially, and if the value was near 1, N was set to exactly 1.0 for computation. To ensure the data were reproducible, each experiment was run at least twice.

#### Mass photometry.

Molecular mass measurement of VPS29C containing Retromer was performed using a Refeyn OneMP mass photometer (Refeyn Ltd) following the manufacturer’s instructions. For the measurement to take place, the purified Retromer in protein buffer at a final concentration of 50 to 100 nM was loaded onto the mass photometer, and 1,000 frames were recorded. The data were examined using Refeyn DiscoverMP software. The molecular mass was calculated using the calibration curves of protein standards with known molecular weight (i.e., 66, 132, and 440 kDa) in the same protein buffer.

#### Biotinylated cyclic peptide pull-down assay.

Streptavidin-based pull-down assay was carried out by adding the purified Retromer fraction with either biotinylated RT-D3 or biotinylated RT-L4 prior to loading into the streptavidin agarose. As a negative control, equal amount of purified Retromer fraction was also loaded directly into the streptavidin agarose without macrocyclic peptide. To avoid precipitation caused by the macrocyclic peptides, the Retromer—peptides mixtures were centrifuged at 17,000 rpm for 20 min at 4 °C prior to adding into the streptavidin agarose. The reaction mixtures were incubated for 2 h at 4 °C. Agarose beads were then washed four times with binding buffer (50 mM Tris-HCl pH 7.5, 200 mM NaCl, 0.1% Triton X-100, 5% glycerol, and 2 mM β-mercaptoethanol) and the samples of beads were analyzed by SDS-PAGE.

#### Alphafold prediction.

To obtain the model of VPS29C and VPS29C containing Retromer, we applied the AlphaFold2 neural network of the open-source ColabFold pipeline. For each modeling experiment, ColabFold was excused using default settings where multiple sequence alignments were generated with MMseqs2. Five models were generated with the top 1 ranked model further relaxed using Amber. Structural alignments and molecular figures were generated using PyMOL and ChimeraX 1.6.1.

#### Phylogenetic analysis of VPS29 sequences.

The full sequence and extended motif (AA 2-29) of Human VPS29C from UniProt (76) (F8VXU5) was used as a query for initial BLAST searches (with default settings) against other metazoans. When BLASTing using only the extended motif, only one nonprimate sequence was found: *Bison bison bison* (XP_010839672.1). To evaluate whether the amino-terminal extension might be present but unannotated in other animal genomes spanning the phylogenetic distance between primates and bison, we performed protein-to-genome searches using miniprot v0.12 (77) against a representative selection of metazoans from the NCBI RefSeq database, using default settings, the –aln and –gff flags, and using human, bison, and subsequently sloth VPS29C sequences as queries. These searches identified VPS29C amino-terminal extensions in range of additional placental mammals, but not in more distantly related lineages. The translated amino acid sequences were aligned using mafft v7.05 (L-INS-i) (78) and visually inspected to find homologous motifs (*SI Appendix*, Fig. S2*A*). Multiple rounds of maximum likelihood tree inference were used to identify homologous sequences using IQTREE v2.2.5 (79), with the best fitting substitution model (-m MFP) and 10,000 ultrafast bootstraps (-B 10,000).

#### Proteomics.

##### Experimental design.

GFP-IP experiments were performed as described above, and following the final wash the beads were processed for mass spectrometry to identify binding partners. All proteomic experiments were performed with isobaric tandem mass tagging (TMT) followed by LC-MS/MS quantitative mass spectrometry. The effects of TMT ratio suppression were minimized by prefractionation of the TMT-labeled pool and use of SPS-MS3-based acquisition to minimize ratio suppression due to coisolation of peptides and, where possible, selecting the labeling set up to minimize any effects of channel bleed-through (80).

##### TMT labeling and high pH reversed-phase chromatography.

GFP-IP samples were reduced (10 mM TCEP, 55 °C for 1 h), alkylated (18.75 mM iodoacetamide, room temperature for 30 min) and then digested from the beads with trypsin (2.5 µg trypsin; 37 °C, overnight). The resulting peptides were labeled with Tandem Mass Tag (TMTpro) 16 plex reagents according to the manufacturer’s protocol (Thermo Fisher Scientific, Loughborough, LE11 5RG, UK) and the labeled samples pooled.

The pooled sample was evaporated to dryness, resuspended in 5% formic acid and then desalted using a SepPak cartridge according to the manufacturer’s instructions (Waters, Milford, MA). Eluate from the SepPak cartridge was again evaporated to dryness and resuspended in buffer A (20 mM ammonium hydroxide, pH 10) prior to fractionation by high pH reversed-phase chromatography using an Ultimate 3000 liquid chromatography system (Thermo Scientific). In brief, the sample was loaded onto an XBridge BEH C18 Column (130 Å, 3.5 µm, 2.1 mm × 150 mm, Waters, UK) in buffer A and peptides eluted with an increasing gradient of buffer B (20 mM Ammonium Hydroxide in acetonitrile, pH 10) from 0 to 95% over 60 min. The resulting fractions (concatenated into 5 in total) were evaporated to dryness and resuspended in 1% formic acid prior to analysis by nano-LC MSMS using an Orbitrap Fusion Tribrid mass spectrometer (Thermo Scientific).

##### Nano-LC mass spectrometry.

High pH RP fractions were further fractionated using an Ultimate 3000 nano-LC system in line with an Orbitrap Fusion Tribrid mass spectrometer (Thermo Scientific). In brief, peptides in 1% (vol/vol) formic acid were injected onto an Acclaim PepMap C18 nano-trap column (Thermo Scientific). After washing with 0.5% (vol/vol) acetonitrile 0.1% (vol/vol) formic acid peptides were resolved on a 250 mm × 75 μm Acclaim PepMap C18 reverse phase analytical column (Thermo Scientific) over a 150 min organic gradient, using 7 gradient segments (1 to 6% solvent B over 1 min, 6 to 15%B over 58 min, 15 to 32%B over 58 min, 32 to 40%B over 5 min, 40 to 90%B over 1 min, held at 90%B for 6 min and then reduced to 1%B over 1 min.) with a flow rate of 300 nL min^−1^. Solvent A was 0.1% formic acid and Solvent B was aqueous 80% acetonitrile in 0.1% formic acid. Peptides were ionized by nano-electrospray ionization at 2.0 kV using a stainless-steel emitter with an internal diameter of 30 μm (Thermo Scientific) and a capillary temperature of 275 °C.

All spectra were acquired using an Orbitrap Fusion Tribrid mass spectrometer controlled by Xcalibur 2.1 software (Thermo Scientific) and operated in data-dependent acquisition mode using an SPS-MS3 workflow. FTMS1 spectra were collected at a resolution of 120 000, with an automatic gain control (AGC) target of 400 000 and a max injection time of 100 ms. Precursors were filtered with a minimum intensity of 5,000, according to charge state (to include charge states 2 to 6) and with monoisotopic peak determination set to peptide. Previously interrogated precursors were excluded using a dynamic window (60 s ±10 ppm). The MS2 precursors were isolated with a quadrupole isolation window of 1.2 m/z. ITMS2 spectra were collected with an AGC target of 10,000, max injection time of 70 ms and CID collision energy of 35%.

For FTMS3 analysis, the Orbitrap was operated at 30,000 resolution with an AGC target of 50 000 and a max injection time of 105 ms. Precursors were fragmented by high energy collision dissociation at a normalized collision energy of 55% to ensure maximal TMT reporter ion yield. Synchronous Precursor Selection (SPS) was enabled to include up to 10 MS2 fragment ions in the FTMS3 scan.

##### Data analysis.

The raw data files were processed and quantified using Proteome Discoverer software v2.4 (Thermo Scientific) and searched against the UniProt Human database (downloaded January 2022; 178486 sequences), an in-house “contaminants” database and against the GFP sequence using the SEQUEST HT algorithm. Peptide precursor mass tolerance was set at 10 ppm, and MS/MS tolerance was set at 0.6 Da. Search criteria included oxidation of methionine (+15.995 Da), acetylation of the protein N terminus (+42.011 Da) and methionine loss plus acetylation of the protein N terminus (−89.03 Da) as variable modifications and carbamidomethylation of cysteine (+57.021 Da) and the addition of the TMTpro mass tag (+304.207 Da) to peptide N termini and lysine as fixed modifications. Searches were performed with full tryptic digestion and a maximum of 2 missed cleavages were allowed. The reverse database search option was enabled and all data was filtered to satisfy false discovery rate of 5%.

#### Statistics.

Further processing and statistical analysis of proteomics data was performed in the R statistical environment (version 4.2). PD2.4 protein grouping was retained, however, the master protein selection was improved with an in-house script which first searches Uniprot for the current status of all protein accessions and updates redirected or obsolete accessions. The script further takes the candidate master proteins for each group, and uses current Uniprot review and annotation status to select the best annotated protein as master protein without loss of identification or quantification quality.

Protein abundances were normalized to GFP, and both raw and normalized abundances were then log_2_-transformed to bring them closer to a normal distribution prior to statistical analysis. Differential protein abundance was estimated using a Welch’s *t* test (*P*-value). Volcano plots were plotted in GraphPad Prism 9 software (LaJolla, CA). Protein–protein interaction network analysis was performed using Metascape 3.5 (81) and visualized using Cytoscape 3.3 software with the Enrichment Map plug-in (82). Dotplots were generated using ProHits-viz (83).

All statistical analysis was performed on data from a minimum of three independent experimental repeats. Unpaired *t* tests were used to compare between the means of two groups, and one- or two-way ANOVA with Dunnett’s or Tukey’s multiple comparisons tests were used to compare the means of more than two groups. Graphs were prepared in GraphPad Prism 9. Individual datapoints represent independent experimental repeats. Graphs are plotted representing the mean value ± SEM for each experimental condition. *n* represents the number of independent experimental repeats. In all graphs, *P < 0.05, **P < 0.01, ***P < 0.001, ****P < 0.0001.

## Supplementary Material

Appendix 01 (PDF)

Dataset S01 (XLSX)

Dataset S02 (XLSX)

Dataset S03 (XLSX)

Dataset S04 (XLSX)

## Data Availability

The mass spectrometry proteomics data have been deposited to the ProteomeXchange Consortium via the PRIDE partner repository with the dataset identifier PXD064959 (https://www.ebi.ac.uk/pride/archive/projects/PXD064959) (84). All other data are included in the manuscript and/or supporting information.
