# Supplementary material for: Identification of a VPS29 isoform with restricted association to Retriever and Retromer accessory proteins through autoinhibition
Source: Proc Natl Acad Sci U S A. 2025 Jun 30;122(27):e2501111122. doi: 10.1073/pnas.2501111122 (PMC12260524; doi:10.1073/pnas.2501111122)
Supplement: Supplementary file 1 — Appendix 01 (PDF) [file pnas.2501111122.sapp.pdf]

PNAS

Supporting Information for

Identification of a VPS29 isoform with restricted association to Retriever and Retromer accessory proteins through auto-inhibition.

James L. Daly[*†], Kai-en Chen[*], Rebeka Butkovič, Qian Guo, Michael D. Healy, Eva Pennink, Georgia Gamble-Strutt, Zara Higham, Edmund R.R. Moody, Philip A. Lewis, Kate J. Heesom, Tom A. Williams, Kirsty J. McMillan[†], Brett M. Collins[†], Peter J. Cullen[†].

* Contributed equally

† Corresponding authors:

James L Daly: James.L.Daly@kcl.ac.uk
Kirsty J McMillan: Kirsty.Mcmillan@liverpool.ac.uk
Brett M Collins: B.Collins@imb.uq.edu.au
Peter J Cullen: Pete.Cullen@bristol.ac.uk

This PDF file includes:

Supporting text
Figures S1 to S6
Tables S1 to S3
Legends for Datasets S1 to S3

Other supporting materials for this manuscript include the following:

Datasets S1 to S3

Supporting Information Text

Full sequence of the *VPS29C* consensus coding sequence, and *VPS29C* PCR products isolated and sequenced from HeLa and HEK293T cells.

Alternating exons are indicated by regular or underlined/italicized text.

VPS29C Coding Nucleotide Sequence (NCBI CCDS ID CCDS73525.1):

atgagcaggtgtgctctcagagggcgggatttggcgcttgcaattgctggaactgtttctttccacgggttgctacgcctctttagg*gctgggcacaga*ttggtgttggtattaggagatctgcacatcccacaccggtgcaacagtttgccagctaaattcaaaaaactcctggtgccaggaaaaattcagcacattctctgcacaggaaacctttgcaccaaagagagttatgactatctcaagactctggctggtgatgttcatattgtgagaggagacttcgatgag*aatctgaattatccagaacagaaagttgtgactgttggacagttcaaaattggtctgatccatggacatcaagttattccatggggagatatggccagcttagccctgttgcagaggcaatttgatgtggacattcttatctcgggacacacacacaaatttgaagcatttgagcatgaaaataaattctacattaatccaggttctgccactggggcatataatgccttggaaac*aaacattattccatcatttgtgttgatggatatccaggcttctacagtggtcacctatgtgtatcagctaattggagatgatgtgaaagtagaacgaatcgaatacaaaaaaccttaa

HeLa cDNA PCR sequence:

ccagagggcgggatttggcgcttgcaattgctggaactgtttctttccacgggttgctacgcctctttagg*gctgggcacaga*ttggtgttggtattaggagatctgcacatcccacaccggtgcaacagtttgccagctaaattcaaaaaactcctggtgccaggaaaaattcagcacattctctgcacaggaaacctttgcaccaaagagagttatgactatctcaagactctggctggtgatgttcatattgtgagaggagacttcgatgag*aatctgaattatccagaacagaaagttgtgactgttggacagttcaaaattggtctgatccatggacatcaagttattccatggggagatatggccagcttagccctgttgcagaggcaatttgatgtggacattcttatctcgggacacacacacaaatttgaagcatttgagcatgaaaataaattctacattaatccaggttctgccactggggcatataatgccttggaaac*aaacattattccatcatttgtgttgatggatatccaggcttctacagtggtcacctatgtgtatcagctaattggagatgatgtgaaagtagaacgaatcgaa

HEK293T cDNA PCR sequence:

cccagagggcgggatttggcgcttgcaattgctggaactgtttctttccacgggttgctacgcctctttagg*gctgggcacaga*ttggtgttggtattaggagatctgcacatcccacaccggtgcaacagtttgccagctaaattcaaaaaactcctggtgccaggaaaaattcagcacattctctgcacaggaaacctttgcaccaaagagagttatgactatctcaagactctggctggtgatgttcatattgtgagaggagacttcgatgag*aatctgaattatccagaacagaaagttgtgactgttggacagttcaaaattggtctgatccatggacatcaagttattccatggggagatatggccagcttagccctgttgcagaggcaatttgatgtggacattcttatctcgggacacacacacaaatttgaagcatttgagcatgaaaataaattctacattaatccaggttctgccactggggcatataatgccttggaaac*aaacattattccatcatttgtgttgatggatatccaggcttctacagtggtcacctatgtgtatcagctaattggagatgatgtgaaagtagaacgaatcg

Fig. S1.

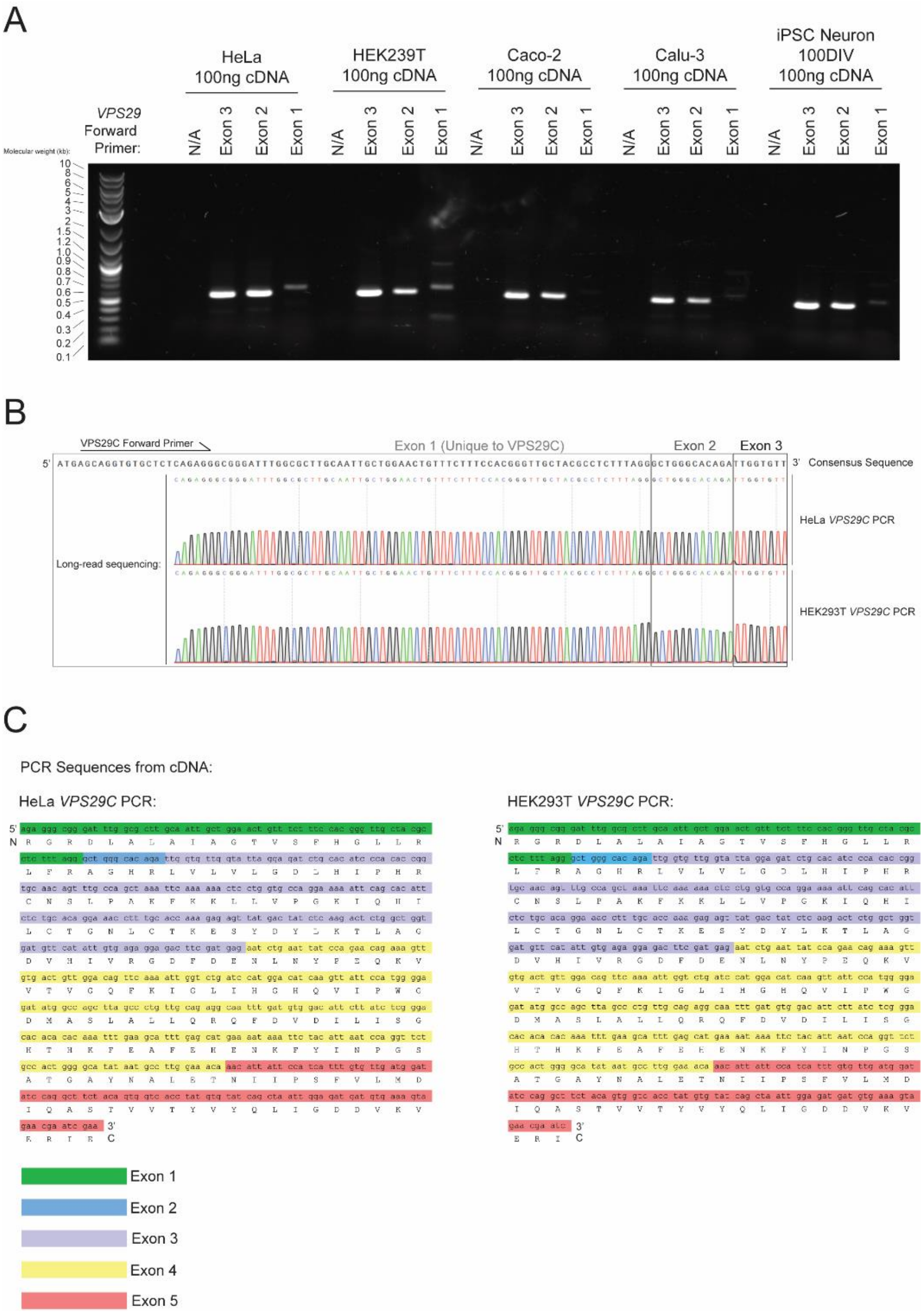

Figure S1 Validation of *VPS29C* expression in human cells

(A) Agarose gel of PCR products generated from HeLa, HEK293T, Caco-2, Calu-3 or cortical neuron induced pluripotent stem cell (iPSC) cDNA. Equal amounts of cDNA were added per condition, and the same 3' reverse primer was used. 5' forward primers corresponding to exon 3, exon 2 and exon 1 amplify products corresponding to the predicted sizes of VPS29A, VPS29B and VPS29C respectively. (B) Alignment of sequencing chromatograms from HeLa and HEK293T PCR products with the consensus 5' end of the *VPS29C* sequence. Exon boundaries and the binding footprint of the 5' end exon 1 primer used to amplify *VPS29C* are indicated, (C) Full sequences of the *VPS29C* PCR products from HeLa and HEK293T cells, coloured by exons. Amino acid sequence is aligned below the nucleotide sequence.

Fig. S2.

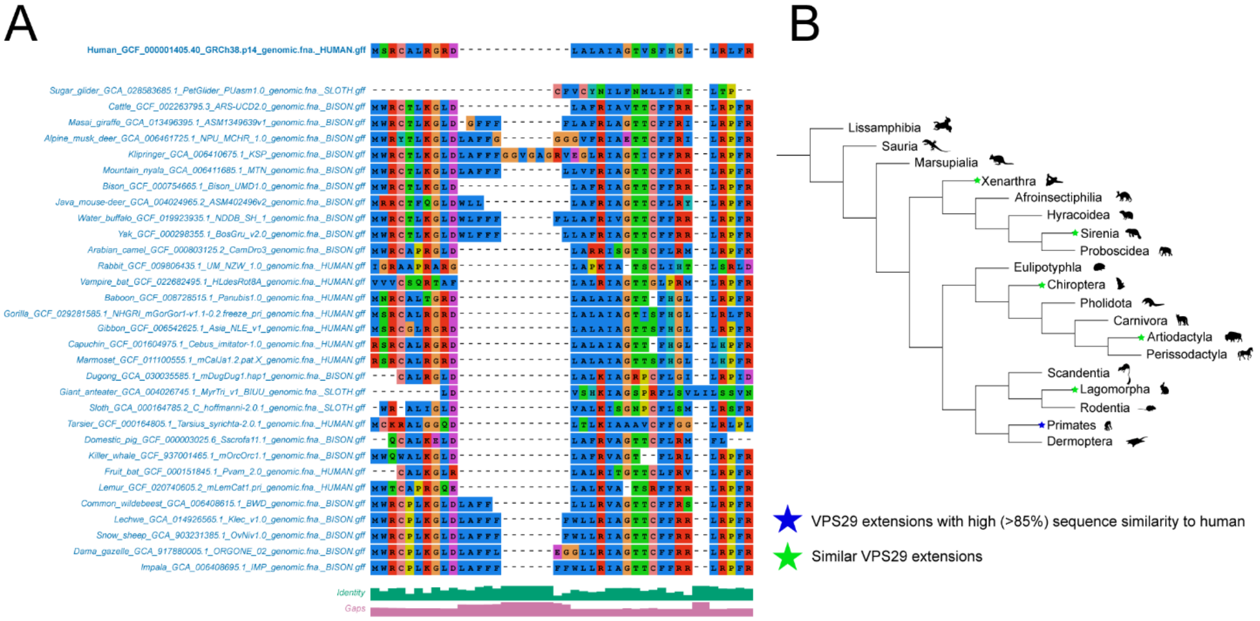

Figure S2. Phylogeny of organisms with an extended VPS29 amino terminus.
(A) Sequence alignment of animal VPS29 extended isoforms. The identifier for each sequence contains the common name of the species encoding the isoform, the genome accession number, and the query sequence (from human, bison or sloth) used to identify the isoform. (B) Schematic phylogenetic tree of animal evolution. Orders containing species with identified VPS29 extended amino-termini are labelled green or blue depending on their degree of sequence similarity to the human VPS29C sequence.

Fig. S3.

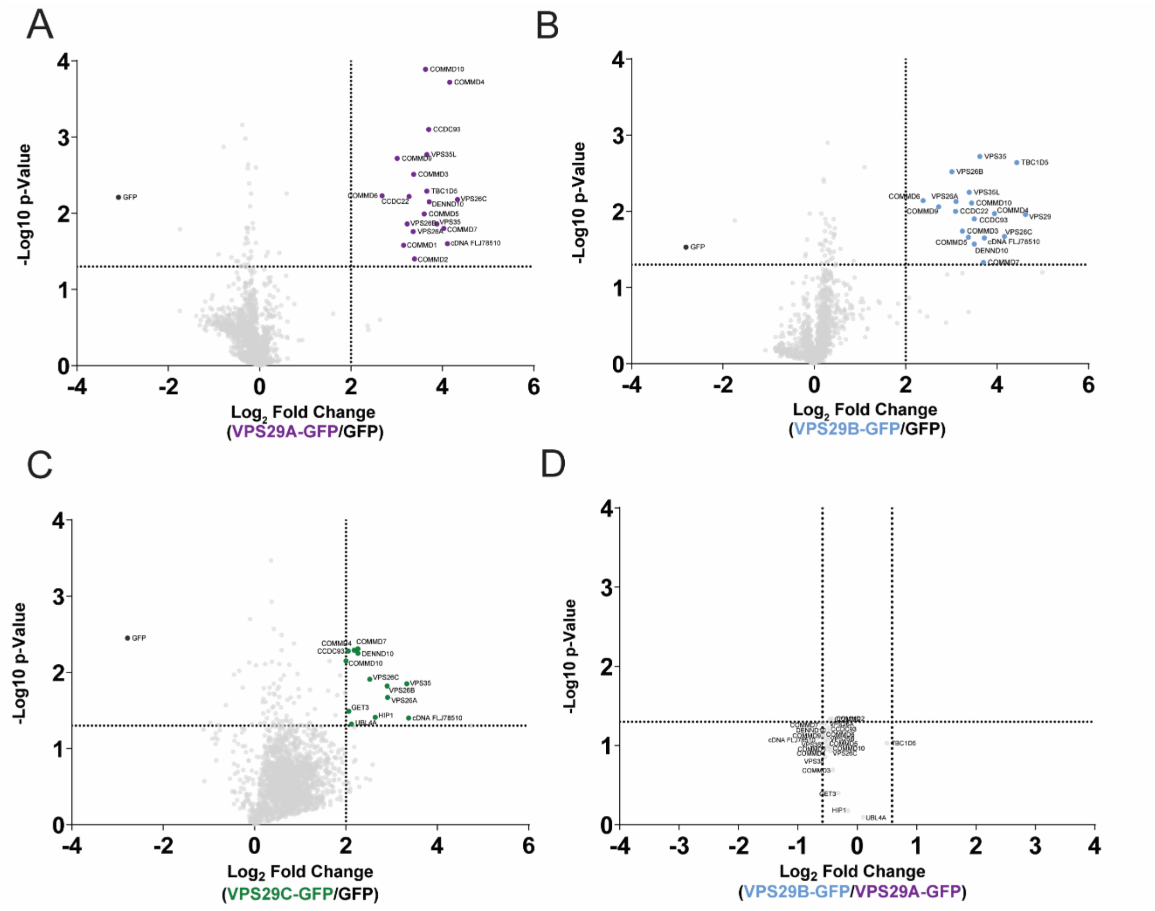

Figure S3. Identification of VPS29 interactors through quantitative proteomics. (A-C) Volcano plots displaying relative enrichment of proteins by VPS29A-GFP (A), VPS29B-GFP (B) and VPS29C-GFP (C) over GFP. Proteins were defined as significant if enrichment exceeded Log_2 fold change > 2, and $p < 0.05$. n = 3 independent experiments. (D) Volcano plot displaying the relative abundances of significant VPS29 interactors between VPS29A and VPS29B following normalisation to GFP expression levels.

Fig. S4.

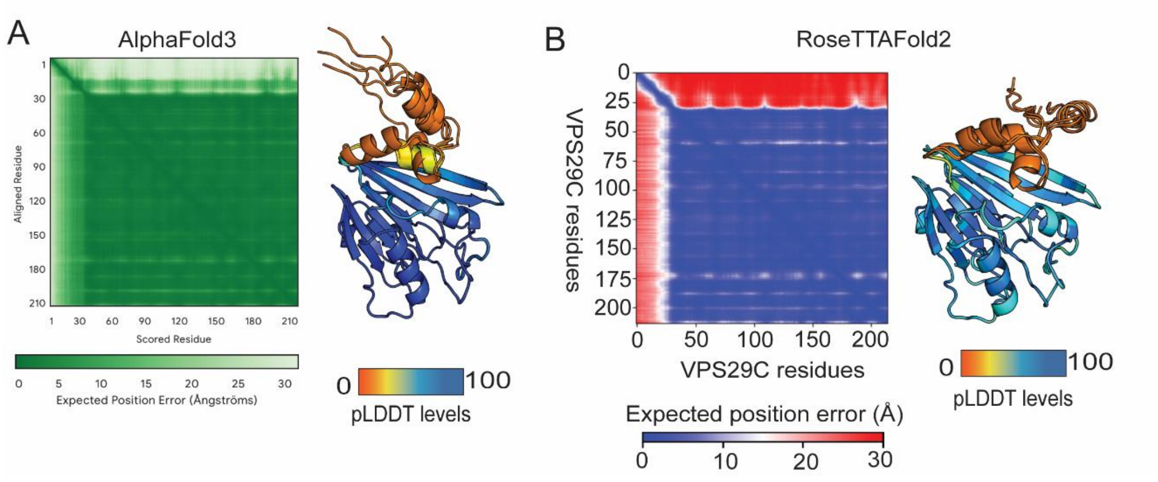

Figure S4: Structural models of VPS29C generated by AlphaFold3 and RoseTTAFold2. (A) PAE plot and cartoon representation of AlphaFold3 and (B) RoseTTAFold2 predicted human VPS29C model. In both cases, top ranked models are overlaid and coloured according to the pLDDT score.

Fig. S5.

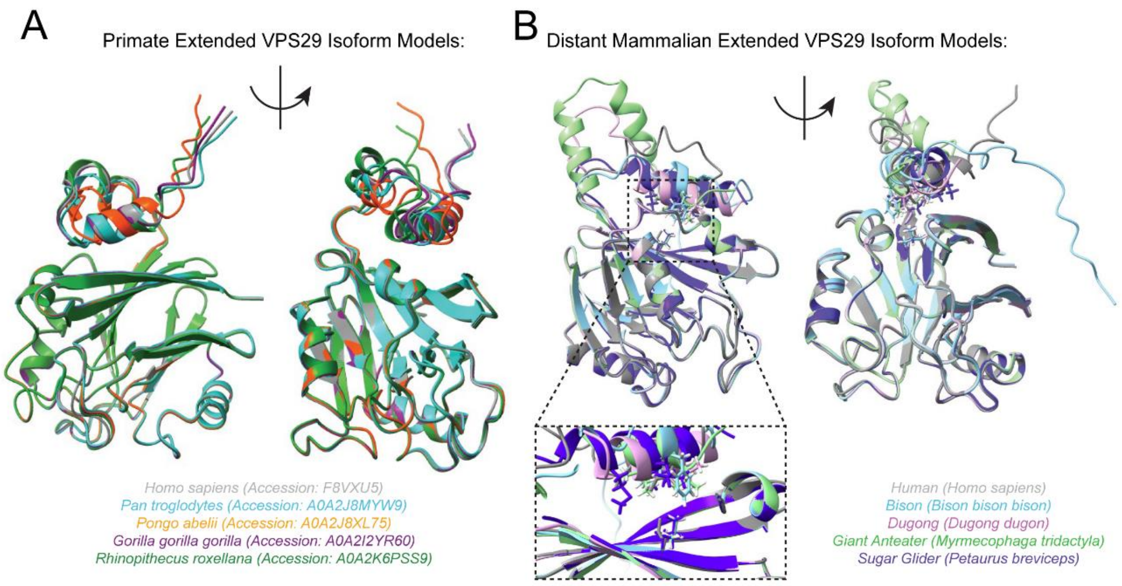

Figure S5. Structural prediction of related VPS29 sequences with extended amino termini. (A) Overlay of AlphaFold2 models of primate VPS29C isoforms, with their corresponding Uniprot accession IDs labelled. (B) Overlay of AlphaFold2 models of human and mammalian VPS29 sequences with extended amino termini. Hydrophobic residues in proximity of Leu-184 or the equivalent Leu residue are shown in stick formation.

Fig. S6.

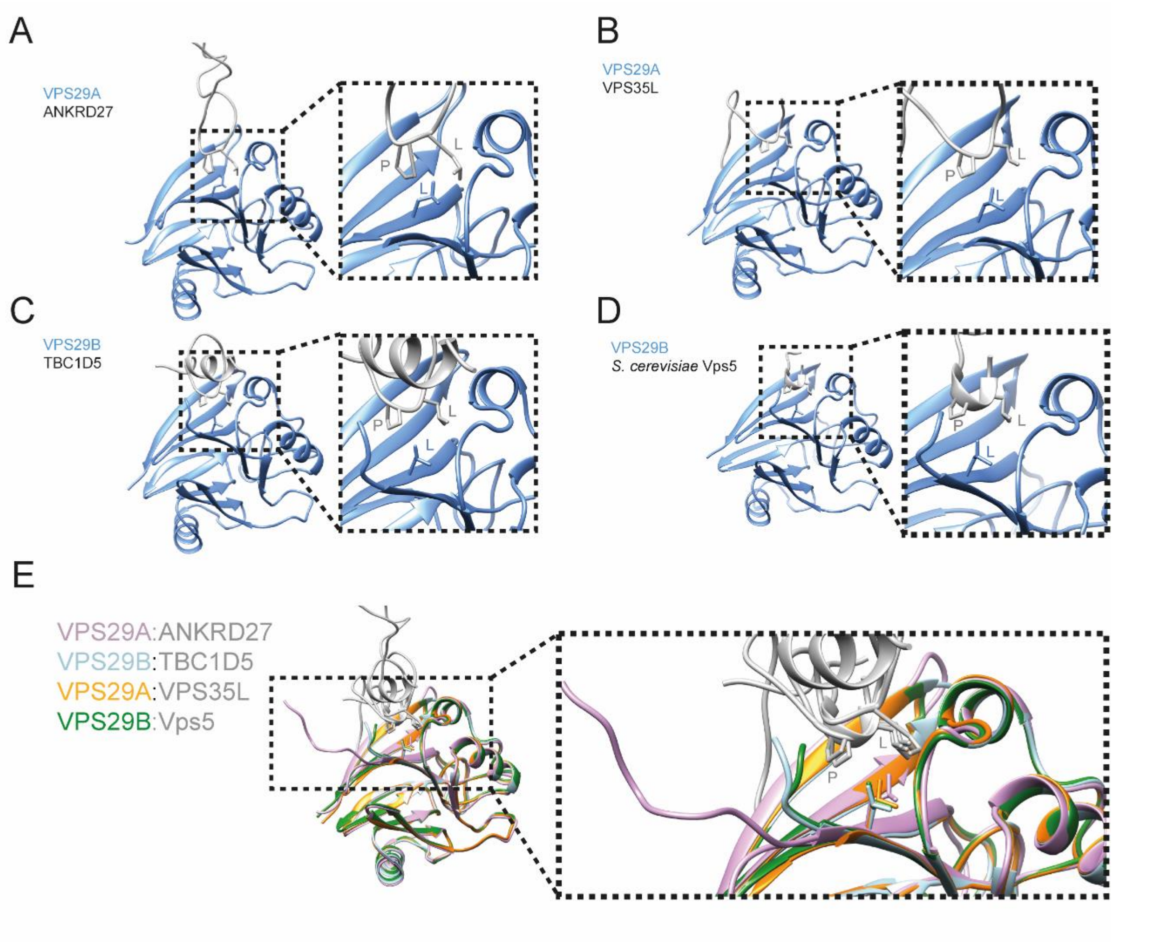

Figure S6. Evolutionary conservation of VPS29 binding to Pro-Leu motif ligands. (A-D) Protein structures of VPS29 isoforms bound to the interacting proteins VARP (PDB: 6TL0), TBC1D5 (PDB: 5GTU), VPS35L (PDB: 8SYL and 8ESE) and *Saccharomyces cerevisiae* Vps5 (PDB: 8FUD). (E) Overlay of structures displayed in (A-D) demonstrating structural similarity of the Pro-Leu motif binding mechanism.

Table S1. Lists of proteins from Supplementary Dataset 2 that were significantly enriched (Log2 fold change > 2 relative to GFP, > 1 unique peptide, p < 0.05) by either VPS29A-GFP, VPS29B-GFP or VPS29C-GFP.

VPS29A-GFP

Gene Name	Log2 Fold Enrichment VPS29A-GFP/GFP	p-Value
VPS26C	4.33	6.60E-03
COMMD4	4.16	1.91E-04
cDNA FLJ78510	4.11	2.51E-02
COMMD7	4.03	1.60E-02
VPS35	3.88	1.38E-02
DENND10	3.71	7.03E-03
CCDC93	3.70	7.95E-04
TBC1D5	3.66	5.11E-03
VPS35L	3.66	1.70E-03
COMMD10	3.63	1.28E-04
COMMD5	3.60	1.03E-02
COMMD2	3.39	3.96E-02
COMMD3	3.37	3.11E-03
VPS26A	3.36	1.75E-02
CCDC22	3.27	5.99E-03
VPS26B	3.23	1.37E-02
COMMD1	3.15	2.66E-02
COMMD9	3.01	1.92E-03
COMMD6	2.68	5.85E-03

VPS29B-GFP

Gene Name	Log2 Fold Enrichment VPS29B-GFP/GFP	p-Value
VPS26C	4.33	6.60E-03
COMMD4	4.16	1.91E-04
cDNA FLJ78510	4.11	2.51E-02
COMMD7	4.03	1.60E-02
VPS35	3.88	1.38E-02
DENND10	3.71	7.03E-03
CCDC93	3.70	7.95E-04
TBC1D5	3.66	5.11E-03

VPS35L	3.66	1.70E-03
COMMD10	3.63	1.28E-04
COMMD5	3.60	1.03E-02
COMMD3	3.37	3.11E-03
VPS26A	3.36	1.75E-02
CCDC22	3.27	5.99E-03
VPS26B	3.23	1.37E-02
COMMD9	3.01	1.92E-03
COMMD6	2.68	5.85E-03

VPS29C-GFP

Gene Name	Log2 Fold Enrichment VPS29C-GFP/GFP	p-Value
cDNA FLJ78510	3.37	3.95E-02
VPS35	3.33	1.41E-02
VPS26A	2.91	2.16E-02
VPS26B	2.90	1.52E-02
HIP1	2.64	3.92E-02
VPS26C	2.52	1.22E-02
COMMD7	2.26	4.90E-03
DENND10	2.26	5.67E-03
COMMD4	2.18	5.12E-03
UBL4A	2.12	4.78E-02
GET3	2.06	3.23E-02
CCDC93	2.05	5.24E-03
COMMD10	2.00	7.04E-03

Table S2. Lists of proteins from Supplementary Table 4 that were significantly enriched or depleted (Log2 fold change +/- 0.5 relative to different VPS29 isoforms, > 1 unique peptide, $p < 0.05$) by either VPS29A-GFP, VPS29B-GFP or VPS29C-GFP.

Gene Name	Protein names	VPS29B-GFP/VPS29C-GFP		VPS29C-GFP/VPS29A-GFP		VPS29C-GFP/VPS29B-GFP		VPS29 GFPC-GFP/VPS29C^{I15D}-GFP	
		LogFC	T-Test	LogFC	T-Test	LogFC	T-Test	LogFC	T-Test
TBC1D5	TBC1 domain family, member 5, isoform CRA_a	0.497241817	0.09239468	-2.752654898	0.004520122	-3.249896715	0.001878954	-0.432968399	0.376882528
COMMD4	COMM domain-containing protein 4	-0.501496928	0.11115222	-2.286482431	0.043041261	-1.784985504	0.032247506	-0.879528121	0.084717104
COMMD5	COMM domain-containing protein 5	-0.502358956	0.08590569	-2.227814899	0.047957051	-1.725455943	0.042094525	-0.870439886	0.123829942
VPS26C	Vacuolar protein sorting-associated protein 26C	-0.445940904	0.11729532	-2.105720223	0.020227809	-1.659779319	0.007135449	-0.682234032	0.032472417
VPS35L	VPS35 endosomal protein-sorting factor-like	-0.543006211	0.08873301	-2.094691528	0.041866303	-1.551685316	0.030008268	-0.68396183	0.074468294
COMMD7	COMM domain-containing protein 7	-0.605166606	0.0528812	-2.062978322	0.000601662	-1.457811715	0.006622315	-0.73508353	0.014241939
COMMD3	COMM domain-containing protein 3	-0.407729643	0.20335271	-2.036020919	0.041043811	-1.628291276	0.018872022	-0.877509549	0.011703244
CCDC93	Coiled-coil domain-containing protein 93	-0.477781279	0.06723788	-1.958528662	0.01291088	-1.480747383	0.004944583	-0.645730999	0.007949039
COMMD10	COMM domain-containing protein 10	-0.461348097	0.10338956	-1.931732477	0.029127037	-1.47038438	0.017599163	-0.689913498	0.034890488
COMMD9	COMM domain-containing protein 9	-0.570444239	0.0727509	-1.857227432	0.045863745	-1.286783192	0.037895688	-0.515112722	0.04946906
COMMD1	COMM domain-containing protein 1	-0.505403687	0.10020075	-1.834978753	0.063279517	-1.329575066	0.052730775	-0.636349038	0.127672636
CCDC22	Coiled-coil domain-containing protein 22	-0.455848734	0.04666166	-1.801602014	0.007403405	-1.34575328	0.004293043	-0.544321805	0.021906003
DENND10	DENN domain-containing protein 10	-0.483760529	0.06054343	-1.753326575	0.002737926	-1.269566046	0.003278916	-0.427325216	0.048442168
COMMD2	COMM domain-containing protein 2	-0.433546877	0.04534118	-1.716166097	0.006746217	-1.28261922	0.03171441	-0.515930095	0.141541461
COMMD6	COMM domain-containing protein 6	-0.573982087	0.07773624	-1.522202561	0.02810343	-0.948220474	0.009629581	-0.403768573	0.002161183
cDNA FLJ78510	cDNA FLJ78510	-0.664939214	0.07939997	-1.039269341	0.247593593	-0.374330127	0.495204335	-0.625914606	0.197128464
VPS35	Vacuolar protein sorting-associated protein 35	-0.540843615	0.13975726	-0.851245196	0.311224707	-0.310401581	0.527496881	-0.665969097	0.164290945
VPS26A	Vacuolar protein sorting-associated protein 26A	-0.543381166	0.05334476	-0.760439258	0.25944749	-0.217058092	0.607786793	-0.590976928	0.169814298
VPS26B	Vacuolar protein sorting-associated protein 26B	-0.494805166	0.0802013	-0.637920806	0.27585295	-0.14311564	0.663730103	-0.459691015	0.165471835
HIP1	Huntingtin-interacting protein 1	-0.160959914	0.66517805	-0.01723908	0.991985372	0.143720834	0.915891921	0.894923319	0.490527908
B4DZC9	Non-specific serine/threonine protein kinase (Fragment)	-0.042403251	0.90138219	0.691614424	0.060313154	0.734017674	0.149422288	-0.263995041	0.49525852
GET3	ATPase GET3	-0.321880444	0.39881782	1.15211831	0.133919062	1.473998754	0.077031641	-0.164768936	0.015795728
UBL4A	Ubiquitin-like protein 4A	0.102914829	0.80482001	1.66520469	0.011434601	1.562289861	0.04973975	-0.494102552	0.059733541

Table S3. Thermodynamic parameters for the binding of Vps29C, Vps35L and cyclic peptides by ITC.

	K_d (μM)	ΔH (kcal/mol)	ΔG (kcal/mol)	-TΔS (kcal/mol)
**GST-VPS29C**				
VPS35L 16-38	>100	-9.54 ± 2.50	-5.26 ± 0.15	4.26 ± 2.04
VPS35L 28-37		No binding detected		
RT-D1 L7E		No binding detected		
RT-D3	13.40 ± 1.67	-2.02 ± 0.71	-6.65 ± 0.07	-5.06 ± 1.44
**GST-VPS29B**				
RT-D3	0.014 ± 0.001	-10.40 ± 0.42	-2.81 ± 0.01	-1.72 ± 1.53

Dataset S1 (separate file). Protein BLAST output of the VPS29C amino terminal extension amino acid sequence. Similar sequences in other organisms are ranked by identity matching.

Dataset S2 (separate file). Raw tandem mass tagging (TMT) proteomics data of GFP, VPS29A-GFP, VPS29B-GFP, VPS29C-GFP and VPS29C^{I15D}-GFP immunoprecipitates. Protein log_2 fold changes are presented as relative to their abundance in the GFP only control condition. *N* = 3 independent experiments.

Dataset S3 (separate file). TMT proteomics data of GFP, VPS29A-GFP, VPS29B-GFP, VPS29C-GFP and VPS29C^{I15D}-GFP immunoprecipitates normalised to GFP levels. Protein log_2 fold changes are presented as relative abundances between VPS29-GFP isoform constructs. *N* = 3 independent experiments.
